# Mitigating black carbon emissions: key drivers in residential usage and coke/brick production

**DOI:** 10.1093/nsr/nwae283

**Published:** 2024-08-14

**Authors:** Jin Li, Yuanzheng Zhang, Shuxiu Zheng, Jinghang Wang, Rong Dai, Wenxiao Zhang, Haoran Xu, Huizhong Shen, Guofeng Shen, Hefa Cheng, Jianmin Ma, Shu Tao

**Affiliations:** College of Urban and Environmental Sciences, Laboratory for Earth Surface Processes and Institute of Carbon Neutrality, Peking University, Beijing 100871, China; College of Urban and Environmental Sciences, Laboratory for Earth Surface Processes and Institute of Carbon Neutrality, Peking University, Beijing 100871, China; College of Urban and Environmental Sciences, Laboratory for Earth Surface Processes and Institute of Carbon Neutrality, Peking University, Beijing 100871, China; College of Urban and Environmental Sciences, Laboratory for Earth Surface Processes and Institute of Carbon Neutrality, Peking University, Beijing 100871, China; College of Urban and Environmental Sciences, Laboratory for Earth Surface Processes and Institute of Carbon Neutrality, Peking University, Beijing 100871, China; College of Urban and Environmental Sciences, Laboratory for Earth Surface Processes and Institute of Carbon Neutrality, Peking University, Beijing 100871, China; College of Urban and Environmental Sciences, Laboratory for Earth Surface Processes and Institute of Carbon Neutrality, Peking University, Beijing 100871, China; School of Environmental Science and Engineering, Southern University of Science and Technology, Shenzhen 518055, China; College of Urban and Environmental Sciences, Laboratory for Earth Surface Processes and Institute of Carbon Neutrality, Peking University, Beijing 100871, China; College of Urban and Environmental Sciences, Laboratory for Earth Surface Processes and Institute of Carbon Neutrality, Peking University, Beijing 100871, China; College of Urban and Environmental Sciences, Laboratory for Earth Surface Processes and Institute of Carbon Neutrality, Peking University, Beijing 100871, China; College of Urban and Environmental Sciences, Laboratory for Earth Surface Processes and Institute of Carbon Neutrality, Peking University, Beijing 100871, China; School of Environmental Science and Engineering, Southern University of Science and Technology, Shenzhen 518055, China

**Keywords:** black carbon, residential sources, coke/brick production, key drivers

## Abstract

Black carbon (BC) is a crucial air pollutant that contributes to short-lived climate forcing and adverse health impacts. BC emissions have rapidly declined over the past three decades and it is important to uncover the major factors behind this decline. Herein, the temporal trends in BC emissions were compiled from 146 detailed sources from 1960 to 2019. Results revealed that the major emission sources were residential solid fuel usage, coke production and brick production. Furthermore, 96.9% of the emission reduction from 3.03 Tg in 1995 to 1.02 Tg in 2019 was attributed to these three sources. It was determined that the transition in residential energy/stove usage, phasing-out of beehive coke ovens and brick kiln upgrading were the most important drivers leading to this reduction and will continue to play a key role in future emission mitigation. In addition, this study identified the need to address emissions from coal used in vegetable greenhouses and the commercial sector, and diesel consumption in on/off-road vehicles.

## Teaser

Black carbon emission inventory for China was fully updated with major drivers affecting the changes quantified.

## INTRODUCTION

Black carbon (BC) is a carbonaceous material composed of polyaromatic sheets and is a major component of fine particulate matter in the air [1,2]. It is considered a short-lived climate forcer that strongly absorbs radiation across the shortwave spectrum, resulting in a warming impact on the climate [3]. Moreover, BC has been shown to have adverse health impacts, including cardiovascular effects, as supported by numerous epidemiological studies [4]. Epidemiological evidence suggests that BC is substantially more toxic than other Particulate Matter (PM) compositions, as demonstrated by a dose–response curve for BC that was four to nine times steeper than that of PM_2.5_ [5].

BC is primarily generated from the incomplete combustion of fossil and biomass fuels [1]. Several bottom-up emission inventories have been developed to quantify BC emissions from various sectors [6–9], providing fundamental data for evaluating the regional and global impacts of BC. According to estimation by the Emissions Database for Global Atmospheric Research, annual anthropogenic BC emission in 2017 from China was 1.11 Tg [6] whereas 1.30 Tg was reported by the Multi-resolution Emission Inventory for China for the same year [9]. The large difference between various inventories primarily stems from data constraints in activities and emission factors (EFs, defined as the ratios between BC emitted and activities conducted). More detailed source information and more accurate data of activities and EFs could help to enhance the reliability of the emission estimation.

In addition, an understanding of the temporal trends in emissions and the major drivers behind these changes is of great importance. This knowledge would allow decision-makers to formulate cost-effective strategies for future abatement actions. Different research groups have reported notable differences in the temporal trends in BC emissions. For example, peak emissions of 2.59 Tg in 1995 [8] and 1.48 Tg in 2013 [6] in China have been reported, with differences in estimates for specific years reaching as high as 345% due to variations in data quality and the level of detail [6,8]. Additionally, the drivers causing these changes in emissions have not been adequately addressed thus far and their quantification could be achieved through structural decomposition analysis [10]. Moreover, BC emissions are mostly reported by sectors of energy production, industry, transportation, residential, agriculture and so on, which hinders a comprehensive interpretation of the effects of detailed drivers on emission changes.

This study aims to update the BC emission inventory for China and to identify and quantify the contribution of major drivers to temporal trends in BC emissions from various sources in China. The inventory was extended to 2019 and the emission types were doubled from 73 in previous inventories to 146, with more detailed and fine sources included [8,11]. A series of new data sets of energy consumption and on-site EF measurements were applied in the updated inventory development. Other information from the literature was also adopted. The inventory has benefitted from the new information and structural decomposition analysis for critical factors driving the long-term variation in BC emissions in the past and coming decades; it has been substantially improved and will provide stronger scientific support for policy implementation in BC pollution mitigation.

## RESULTS AND DISCUSSION

### Residential and coke/brick production predominantly contributed to BC emissions and their temporal trends

Based on the extensively updated data on emission activities and EFs (see ‘Methods’), the annual BC emissions from all sources in 2019 in China were calculated as 1.02 Tg. This calculation involved the quantification of emissions from 146 individual sources, providing a much more detailed perspective than other inventories. The results are compared with two inventories published previously [8,11], showing significant improvements in the updated inventory in many aspects, as shown in Fig. S1. The updated inventory covers a period of 60 years from 1960 to 2019 with monthly temporal resolution and daily emissions can be generated if required. Residential emissions, considered the most important sources, were distinguished from commercial emissions and categorized into 32 detailed activity/fuel types (see ‘Methods’). Moreover, both the activity strengths and the EFs of most sources were thoroughly updated, incorporating recent on-site measurements and literature data (see ‘Methods’). These updates resulted in a substantial improvement in data quality regarding annual emissions, temporal trends and source profiles, particularly regarding the source types. For example, the BC emissions from residential/commercial usage and coke production in 2017 were estimated as 52% higher and 41% lower, respectively, than those previously reported [8]. It is reasonable to expect that the uncertainty in the estimation was reduced to a certain extent by distinguishing detailed source types with different EFs and including new data [1,12]. In addition, detailed source profiles hold considerable value for decision-makers.

**Figure 1.**
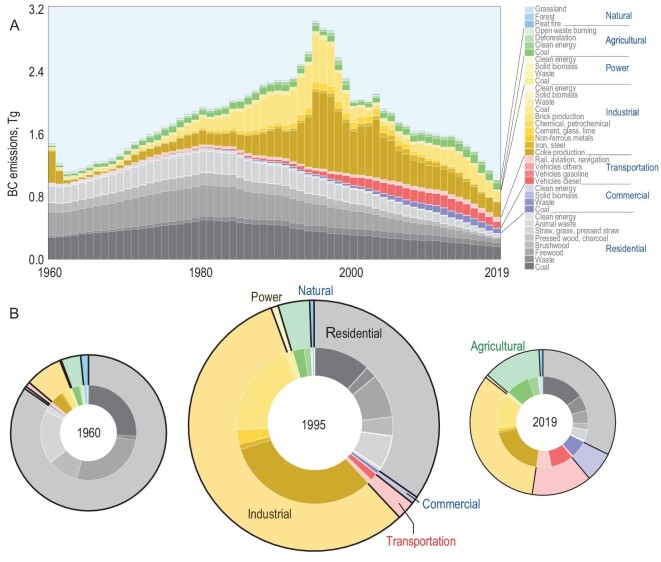
(A) Temporal trends in black carbon (BC) emissions from 37 major source types across seven sectors from 1960 to 2019. (B) Source profiles in 1962, 1995 and 2019 are shown as ring charts, in which the sizes (areas) of the rings are proportional to the total annual emissions.

From 1960 to 2019, both the annual emissions and the source profiles exhibited significant changes, as shown in Fig. 1, which presents the temporal variation in BC emissions from major sources. The 146 source types used in this study were grouped into 37 major types and only a dozen or so key sources contributed enough to be visible in the figure. Detailed data can be found in Table S1. Source profiles of typical years, namely 1962, 1995 and 2019, are shown as three-ring charts, with the full areas of the rings being proportional to the annual emissions. The total annual emission was 1.11 (0.90–1.41 as 50% uncertainty interval) Tg in 1962, increased monotonically to a peak value of 3.03 (1.92–5.15) Tg in 1995 and declined thereafter to 1.02 (0.75–1.52) Tg in 2019. A small but distinct peak observed in around 1960 was attributed to the operation of numerous beehive coke ovens during the period known as the Great Leap Forwards [13]. In 1962, residential solid fuel combustion dominated the BC emission sources, accounting for 83.2% of the total emissions that year. Within the residential sector, fuelwood, coal and straw consumption contributed 36.8%, 26.2% and 18.2% of the total emissions, respectively. However, by the emission peak year of 1995, the relative contribution of the residential sector had significantly declined to 34.7%, while the industrial sector's contribution had risen from 7.8% in 1962 to 56.4% in 1995, primarily due to the substantial increase in coke (32.0%) and brick (19.5%) production. By 2019, the most significant sources of BC emissions were coke production (18.7%), residential coal consumption (15.4%), brick kilns (12.2%), residential biomass fuels (10.8%) and diesel vehicles (8.0%). In sum, the predominant contributors were the consumption of solid fuels in the residential sector and industrial coke and brick production. These three sources accounted for 89.4%, 83.6% and 57.4% of the total emissions in 1962, 1995 and 2019, respectively. Although the transportation and agricultural sectors contributed relatively little to the total BC emissions, their shares have increased in recent years due to the expansion of the motor vehicle fleet and coal consumption in vegetable greenhouses [14,15], despite a substantial reduction in residential and industrial emissions. The relative contributions of these two sources increased from 4.1% in 1995 to 16.7% in 2019.

Although the temporal trend in BC emissions cannot be validated directly, the general decline trend can still be confirmed by using field observations of BC concentrations in ambient air. Based on a multicity monitoring campaign, it was reported that BC concentrations in ambient air decreased by 15% in Beijing, 17% in Tianjin, 40% in Nanjing and 50% in Xi'an from 2013 to 2017, respectively [16], during which the total emissions decreased by 20.2% as a national average. In addition, the rapid reduction in BC emissions in China has been also confirmed by the observed reduction in BC concentration in Fukue Island, Japan, where the majority of BC aerosols could be sourced to mainland China [17].

**Figure 2.**
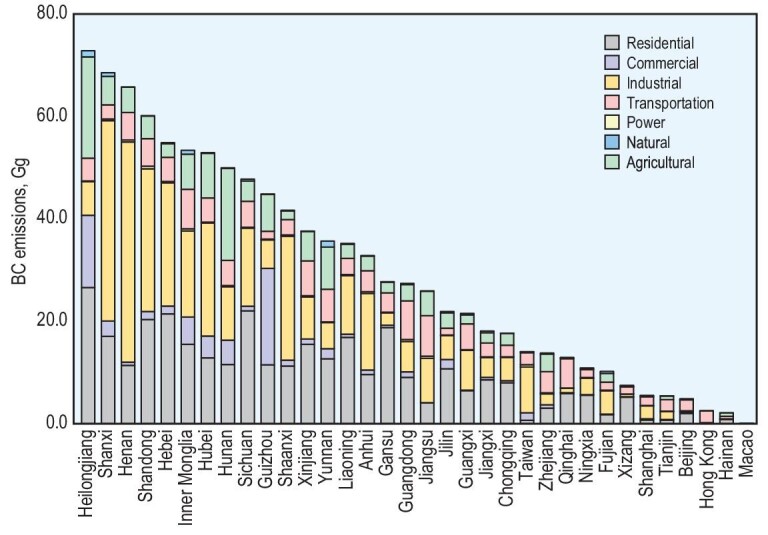
Annual black carbon emissions from seven sectors for each province in China in 2019 (including Hong Kong, Macao and Taiwan province).

Over the years, BC emissions in China have exhibited extensive spatial variation owing to diverse socio-economic conditions. To illustrate this, Fig. 2 shows the annual BC emissions from seven sectors for each province in China in 2019. The spatial pattern of residential BC emissions is closely associated with rural population density, living conditions and climate. Higher residential emissions are observed in densely populated eastern regions with less developed areas, where solid fuels are commonly used [18]. Additionally, colder regions with greater winter heating needs exhibit elevated residential emissions. In 2019, the top five provinces for coke production (Shanxi, Shaanxi, Shandong, Hebei and Inner Mongolia) contributed ∼60% of the national total coke production [19]. Consequently, these provinces accounted for 39.2% of the total BC emissions from the industrial sector. However, the spatial pattern of BC emissions from brick production correlates more closely with the rural population density, as the demand for brick is relatively consistent nationwide and the long-range shipment of bricks is economically impractical [20]. Other sources with relatively high BC emissions include areas with strong commercial emissions from coal consumption in Guizhou and Heilongjiang, where commercial coal usage is prevalent [19], and regions with intensive agricultural emissions resulting from coal use in vegetable production in Hunan and Heilongjiang [19]. Major metropolitan areas experience high emissions due to heavy traffic volume in these cities.

### Residential BC emissions were predominantly reduced through energy and stove switching and urbanization

As discussed above, residential emissions ranked highest among the seven sectors all the time, particularly in the early years. Residential emissions from various solid fuels were the most significant contributors to both total emissions and the overall temporal trend. Prior to 1980, the increase in total BC emissions was primarily driven by increased residential emissions. By differentiating the emissions from the residential and commercial sectors, which were previously combined, it was discovered that residential emissions were much more important than commercial emissions in the past. However, the relative contribution of commercial emissions has increased in recent decades due to emission reduction from residential and industrial sources and rapid expansion of the catering industry [19]. The residential domination in BC emissions lasted for about two decades during the 1960s and 1970s, when residential emissions were generally proportional to the total population. After 1980, the Chinese economy began to take off and rapid socio-economic transformations occurred, significantly impacting residential energy use and stove types [21], consequently leading to a continuous decrease in BC emissions despite population growth. As a result of these changes, residential emissions decreased significantly, with a relative contribution of 84.6% in 1962, declining to 34.7% in 1995 and further decreasing to 32.2% in 2019. In China, there were distinct differences in residential energy types used between urban and rural areas. Traditional coal cooking/heating stoves have been popular in Chinese cities throughout history. However, since 1965, cylindered liquefied petroleum gas has been promoted as a replacement for coal stoves in cooking [22] and pipelined natural gas has been widely introduced in cities across the country since the end of the last century [23]. As a result, the penetration rate of these gases increased from 14.4% in 1978 to 82.1% in 2005 and 97.3% in 2019 in cities [24]. Simultaneously, the coverage of centralized heating systems in northern China expanded from 1.9% in 1981 to 52.1% in 2019 [15,24], leading to substantial reductions in residential BC emissions in cities. In rural areas, both coal and biomass fuels have been extensively used in the past [18]. Two major factors driving the reduction in BC emissions in the rural residential sector are energy transitions and stove switching [21]. As living conditions improved, solid fuels used in rural areas for cooking and heating have gradually been replaced by electricity and liquefied petroleum gas, resulting in significant reductions in BC emissions [18]. It has been revealed that the shift from solid fuels to cleaner energy sources for rural residential cooking primarily depends on affordability and the increasing trend in clean energy used for cooking can be predicted using per-capita income as a proxy for living conditions [18]. At the same time, rural residential stoves have transitioned from open stoves to traditional, energy-saving and clean stoves [21], accompanied by a reduction in BC emissions. For example, the EFs of BC for traditional stoves burning firewood (56.4 ± 23.7 mg/MJ) are significantly higher than those for clean stoves burning wood pellets (1.99 ± 1.63 mg/MJ) [25].

In addition to the energy/stove transition, rapid urbanization led to massive migrations of millions of rural residents to cities after the 1980s [15]. In general, urbanization has resulted in a reduction in BC emissions because biomass fuels are inaccessible in cities [26]. Although urbanization has led to increased BC emissions in urban areas, primarily due to the rural-to-urban migrants’ using more coal for heating, this increment is incomparable to the reduced population and BC emissions in rural China [26], leading to an overall emission reduction driven by urbanization. Another positive factor influencing BC emissions is population expansion with heating demand. Historically, the so-called Qin–Huai line, running along the Qin Mountains and the Huai River, was officially designated in the early 1950s to divide regions into heating and non-heating areas [27]. To the north of this line and in Qinghai and Xizang in the west, heating facilities were predefined in the Code of Building Design in China [28] and infrastructure and government subsidies were only provided in these heating regions [29]. The division remained relatively unchanged until the 1990s, when living conditions began to improve rapidly. Since the 1990s, some residents south of this line have begun to heat their homes when winter temperatures drop to nearly 0°C, provided they can afford the cost [15,30]. According to a nationwide survey conducted in 2012, the percentage of the rural population who were heating their homes increased from 32.2% in 1992 to 55.6% in 2012 [30]. Similarly, new buildings with individual or centralized heating facilities have been constructed in traditionally non-heating regions [24]. Concurrently, the heating periods are extended in traditional heating regions, resulting in increased fuel consumption and BC emissions. The expansion of the heating population is likely to continue as economic growth persists in the future.

**Figure 3.**
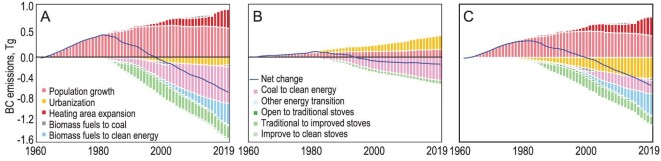
Major drivers affecting BC emissions from the residential sector, displaying the cumulative contributions of these drivers from 1960 to 2019. The results are shown for (A) the entire country, (B) urban areas and (C) rural areas. In addition to the 10 major drivers, the net contributions are represented by the differences between the total positive and total negative contributions.

The above-mentioned factors influencing BC emissions from the residential sector were quantified and the results are shown in Fig. 3 as the cumulative contributions of major drivers to the temporal trend in the BC emissions from the residential sector from 1960 to 2019. Additionally, the results are presented separately for rural and urban areas. The net contributions are represented by the difference between the total positive and total negative contributions. Nationwide, the major positive drivers were population growth and heating population (area) expansion. The dominant negative factors were residential energy mix transition and stove switching, particularly the transition from coal and biomass fuels to cleaner energy (oil&gas) sources. The upgrade from traditional to improved stoves also made a remarkable contribution to emission reduction. While most factors influenced both rural and urban areas in the same direction, urbanization had a positive effect in urban areas and a negative effect in rural areas, leading to slightly negative net effects on the overall trend. Accumulatively, BC emissions from the residential sector decreased by 0.62 Tg from 1960 to 2019, with energy switching (−1.13 Tg; −182.3%), population growth (0.55 Tg; 88.7%), stove upgrading (−0.29 Tg; −46.7%), urbanization (−0.14Tg; −22.6%) and expansion of the heating population (0.35 Tg; 56.5%) playing a predominant role in the total change over the 60 years. The population contribution growth leveled off in around the mid-1990s whereas the influence of the energy/stove transition has steadily increased since then. Therefore, the accumulative net contributions increased during the first three decades and turned negative afterward. Notably, the changes in rural areas are much larger than those in urban areas, with the emissions in rural areas initially being much higher than those in urban areas.

### Banning beehive coke ovens led to a substantial reduction in industrial BC emissions

BC emissions from >40 industrial sources were quantified and classified into emissions from industrial combustion of various fuels and emissions directly from various fuel-free processes. Due to the dominance of fugitive emissions [31], coke production ranked as the highest contributor to industrial BC emissions among all quantified sources. Consequently, temporal changes in BC emissions from industrial sources were predominantly driven by changes in emissions from coke production.

China has consistently held the top position as the global producer and supplier of metallurgical coke for many years [14]. In 2019, total coke production in China reached 485 million tons, accounting for 69% of the global total [14]. It is well established that coke production is associated with substantial emissions of various air pollutants, including BC [32]. These emissions primarily stem from coal-loading, coking, coke discharge and coke-quenching processes during the operation of coke batteries [31]. BC emissions from these processes are largely fugitive, escaping through battery doors, hatches and standpipes. Due to technical challenges in implementing end-of-pipe dust removal for unorganized sources [31], the EFs of BC for fugitive emissions are significantly higher than those emitted through chimneys [33]. Consequently, the overall EFs for coke ovens are much higher than those for most other industrial processes [32,34]. In fact, the relative contributions of coke production to total industrial BC emissions increased from 52.1% in 1962 to 56.8% in 1995.

**Figure 4.**
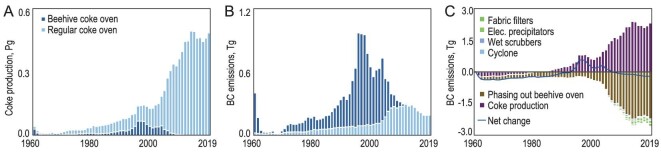
(A) Temporal trends in coke production and (B) BC emissions from coke ovens in China. The contributions of both regular industrial-scale coke batteries and beehive coke ovens are shown separately. (C) Major drivers affecting BC emissions from coke production are also shown as accumulative contributions of individual drivers from 1960 to 2019. In addition to the six major drivers identified, net contributions are represented by the difference between the total positive and negative contributions.

In China's history, coke was produced using regular industrial-scale coke batteries or crude beehive coke ovens. Beehive coke ovens were extensively built and operated to meet the rapidly increasing demand, which could not be achieved quickly enough through regular coke batteries, particularly in the 1990s and 2000s [35]. These beehive coke ovens were typically homemade by local farmers at a very low cost [35]. Due to their long-lasting coking process, during which leakage cannot be fully insulated, large quantities of BC are released in an uncontrolled manner from beehive coke ovens. Consequently, the EFs of BC for beehive coke ovens are approximately an order of magnitude higher than those from industrial-scale coke batteries [32]. When the strong emissions without any control measures were recognized, the beehive coke ovens were officially banned by the Coal Law in 1996 [36]. However, full implementation of the ban did not occur until 2011 [35]. Figure 4A and B shows the temporal trends in coke production and associated BC emissions, distinguishing between industrial-scale coke batteries and beehive coke ovens using stacked bar charts. Total coke production increased from the early 1960s to 2013, with particularly high growth rates from 2003 to 2013, corresponding to the rapid increase in steel production [37]. Although the total coke production from beehive coke ovens over the 60 years accounted for only 10.1% of the total production, the contribution to total BC emissions (11.8 Tg) from total coke production was as high as 65.3% due to the extremely high EFs. Consequently, the overall temporal trends in coke production and BC emissions exhibited notable differences. The former depended primarily on industrial-scale coke production whereas beehive coke ovens predominantly governed the latter. During its peak year in 1995, 48.9% of annual coke production and 90.7% of annual BC emissions associated with coke production were attributable to beehive coke ovens [14,35]. BC emission from beehive coke production was eliminated when they were finally banned in 2011 [35]. A small but sharp peak in BC emissions can be observed in around 1960 (data are not available before 1960) due to a massive campaign of iron-smelting using backyard furnaces and beehive coke ovens during the Great Leap Forwards [13]. In 1960, BC emissions from industrial sources amounted to 0.45 Tg, with 85.0% from the beehive coke ovens. The emission dropped to 0.09 Tg in 1962 at the end of the campaign.

The major drivers behind the changes in BC emissions from coke production were quantified and are shown in Fig. 4C in cumulative terms. The key drivers include coke production, the phasing-out of beehive coke ovens and various abatement measures such as dust removal technologies using cyclones, wet scrubbers, electrostatic precipitators and fabric filters. The rapid change in BC emissions began in the 1990s. The two key drivers that dominated the overall trend were the increase in total coke production and the phasing-out of beehive coke ovens. These two drivers worked in opposite directions to a similar extent, resulting in relatively small net contributions to the total emission variation. It is reasonable to expect that, if the beehive coke ovens had not been banned, the total BC emissions from coke production would have surged to ∼2.63 Tg due to the rapid increase in demand [35]. In comparison, the installation of dust removal facilities had a much smaller impact, leading to a slight decrease in total emissions. Differently from emissions from point sources, the mitigation of fugitive emissions from coke batteries poses technical challenges [31], despite efforts to improve dust removal efficiency through the installation of sophisticated and relatively expensive facilities such as dust deflectors, enclosed belt conveyors and dust hoods [38]. From the peak emission year of 1995 to 2019, the installation of dust removal facilities led to a total reduction of 0.27 Tg in BC emissions, which is significantly smaller than the reduction achieved by banning beehive coke ovens. Nonetheless, coke production still accounted for 18.8% of total anthropogenic emissions in 2019, highlighting the high potential for future emission mitigation.

### BC emissions from brick production were predominantly reduced due to kiln upgrading

Similarly to coke production, China also ranks at the top in clay brick production and consumption, accounting for approximately half of the total output and usage worldwide [39,40]. Brick kilns, such as those used in coke production, were the second-largest BC emission source in the industrial sector. The main reason for their dominant contribution to BC emissions is the fugitive emissions from the loosely observed kiln roofs that contain the coal slag [41]. On-site measurements for 18 representative kilns in China revealed that the EFs of BC from fugitive sources (0.1 ± 0.06 g/kg coal) were much higher than those from stacks (0.029 ± 0.022 g/kg coal) [41]. The inclusion of fugitive emissions significantly improved the overall estimation for this particular source compared with previous studies.

**Figure 5.**
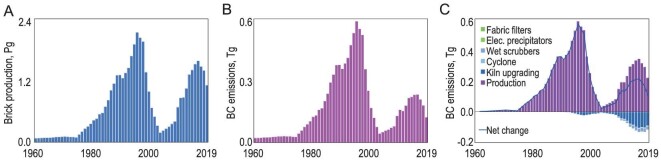
(A) Temporal trends in brick production and (B) BC emissions from brick kilns in China. (C) Major drivers affecting BC emissions from brick production are shown as cumulative contributions of individual drivers from 1960 to 2019. In addition to the six major drivers, the net contributions are represented by the difference between the total positive and negative contributions.

Most clay bricks in China were made using coal-fired kilns and Fig. 5A and B shows the temporal trends in total brick production and associated BC emissions from 1960 to 2019. There were two general peaks in production in around 1995 and 2016. The first wave began in the mid-1970s to meet the booming demand for infrastructure driven by rapid economic development and improved living conditions [15]. Production reached a peak of 2138 million tons in 1995 and then dropped rapidly due to the imposed ban on traditional clay brick kilns [42,43]. Since 2003, the banned solid bricks have been replaced by hollow bricks, leading to a surge in production and the second wave, which reached a peak of 1584 million tons in 2016. However, it appears that the demand dropped after that year. Similarly, there were two similar waves in BC emissions from brick production. The significant difference in the overall temporal pattern between the production and emissions was the relatively low emissions during the second wave. This difference was caused by reduced EFs in recent years due to brick kiln upgrading and the installation of end-of-pipe dust removal facilities [44]. Because BC is predominantly associated with PM_2.5_ [45], the removal efficiencies of end-of-pipe BC removal are similar to those for PM_2.5_.

The effects of major drivers of BC emissions from this source were identified and quantified, as shown in Fig. 5C. In addition to the positive drivers of brick production (coal consumption) on BC emissions from brick sintering, several factors contributed to emission reduction. The most important negative driver was the replacement of annular kilns with tunnel kilns, which has predominantly contributed to the reduction in BC emissions from clay brick production since the beginning of the century. In comparison, dust removal had a much smaller impact on emission reduction, as end-of-pipe facilities cannot effectively mitigate strong BC emissions from fugitive sources. From 1960 to 2019, the increase in clay brick production led to an increase of 0.22 Tg in BC emissions, while the development of tunnel kilns and dust removal efforts resulted in reductions of 0.09 and 0.02 Tg in BC emissions, respectively.

### Policy implication

Compared with previous inventories that were developed by us and other groups [6,8,9,11], a number of improvements were achieved in this study by further categorizing emission sources, including more data on the activities, EFs and penetration rates of abatement technologies. Among many sectors, residential and commercial emissions were separated and the navigation sources were divided into ocean ships, inland ships and small fishing boats, which vary remarkably [46,47]. The total number of sources was doubled from 73 in the previous inventory to 146 in the updated inventory. A large amount of valuable data such as fugitive emissions from brick kilns [41] were included. All these updates help in improving both the precision and the usefulness of the inventory.

**Figure 6.**
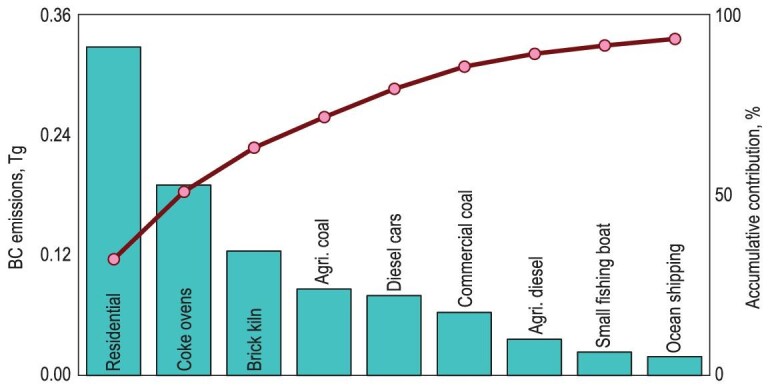
BC emissions from the top nine sources in 2019. Cumulative contributions of these sources to total anthropogenic emissions are also shown.

The three major sources of residential solid fuels, coke ovens and brick kilns accounted for an average of 78.9% of the total anthropogenic BC emissions from 1960 to 2019. The dominance of these sources is largely attributed to the challenging nature of the mitigation of fugitive emissions from these specific sources. Similarly, the overall reduction in total anthropogenic BC emissions was primarily driven by the temporal change in the emissions from these three sources. Collectively, the transition in residential energy/stoves, the phasing-out of beehive coke ovens and the upgrading of brick kilns contributed to a reduction of 3.74 Tg in BC emissions from 1960 to 2019, accounting for 838% of the total accumulative emission reduction from all anthropogenic sources. However, despite these efforts, the three major emission sources remained at the top of the emission list, accounting for 57.4% of the total emissions in 2019. Figure 6 shows the top nine BC emission sources in 2019, which collectively contributed to 93.2% of the total BC emissions that year and need to be addressed in future BC emission abatement strategies.

In the residential sector, both coal and biomass fuels play equally important roles in BC emissions. If the remaining coal and biomass fuels used for rural residential cooking and heating could be reduced by half, then it would result in the elimination of 0.12 Tg of BC emissions, which is equivalent to 11.5% of the total anthropogenic emissions in 2019. In reality, a successful clean heating campaign was implemented in North China Plain from 2017 to 2021, leading to the replacement of >70% of the solid fuels used for residential heating by pipelined natural gas or electricity in that region [48]. The campaign is currently being expanded to encompass all of northern China [48], showcasing its high potential for future BC emission reduction. Technically, the control of emissions in coke and brick production is relatively challenging owing to their non-organized feature.

In addition to the three top primary sources mentioned earlier, attention needs to be given to BC emissions from coal used in vegetable greenhouses, the catering industry and diesel consumed by on- and off-road vehicles. Among the nine sources highlighted, agricultural emissions from vegetable greenhouses have contributed consistently to BC emissions over the past 60 years. As the dominant source in the agriculture sector, bituminous coal is combusted in greenhouses in the northern regions to cultivate off-season vegetables [49]. The usage of vegetable greenhouses was promoted by the introduction of plastic film in around 1960 and prefabricated thin-walled steel pipes in the 1980s [50,51]. The contribution from this sector to total BC emissions increased from 2.4% in 1995 to 8.5% in 2019, partially due to the reduced emissions from other sources. Currently, there are no specific regulations applicable in controlling air pollutant emissions from vegetable greenhouses, except for a related standard on Green Food-Environmental Quality for Production Areas (NY/T 391–2021) [52]. Future action needs to be taken to mitigate emissions from this source. Compared with those of the residential and industrial sectors, the contribution of motor vehicles to the overall BC emissions before the 1990s can almost be ignored. However, in addition to the rapid reduction in emissions from the residential sector and coke and brick production, the relative contribution from motor vehicles, mostly from diesel vehicles, to the total emissions actually increased, despite the tightening-up of the emission standards [53]. Currently, heavy-duty diesel vehicles are considered another important BC emission source. In the peak year of 2012, BC emissions from this source reached 0.15 Tg, accounting for 9.5% of the total emissions. Due to positive contributions such as an expanding vehicle fleet and negative contributions such as exhaust control promotion, emissions from motor vehicles increased until 2012 and decreased thereafter, despite the continuous growth in the vehicle fleet [15]. By 2019, emissions had decreased to 0.08 Tg, accounting for 7.8% of the total emissions (8.2% from all on-road diesel vehicles) and further reduction in the future is expected.

## METHODS

### Detailed emissions

A comprehensive approach was taken to develop detailed source profiles to support decision-making in BC emission abatement. Therefore, a total of 146 detailed source types across seven sectors (power generation, industry, transportation, agriculture, residential, commercial and natural) and various fuel/activity types (coal, oil, gas, waste, biomass and industrial processes) were included in the analysis. The complete list of detailed source types can be found in the Table S1. Several updates were made, particularly in the residential and commercial sectors, which are now considered separate entities. Emissions from crop residues were quantified for eight specific types: maize stalk, corn cob, wheat, rice, sugar cane, soybean, cotton and others. Additionally, important sources such as coal consumption in vegetable greenhouses were newly added. In addition to the detailed source types, the spatio-temporal variation in emissions was disaggregated at a resolution of 0.1° every month.

### Data sources

To compile the bottom-up emission inventory, most energy data were obtained from the International Energy Agency [14], supplemented by relevant local data from several important sources. Detailed data on rural residential energy consumption, which is a crucial BC source, were derived from two nationwide face-to-face surveys, with daily biomass fuel consumption weighting covering 34 489 households in 2012 and 56 556 households in 2017 [18,54]. Historical emission data of beehive coke ovens were extracted from remote sensing images that captured spatio-temporal variations [35]. Similarly, EFs were extensively updated, incorporating newly published data from the literature, which were collected, cleaned and integrated into the existing database [55]. Several hundreds of on-site measured EFs for residential sources [56] and more than a dozen field-measured EFs for fugitive emissions from brick kilns [41] were incorporated. The most important data updates are listed in Table S2, with particular emphasis on the three primary sources: residential usage, coke production and brick production.

### Spatio-temporal disaggregation

Total annual emissions were disaggregated into 0.1° × 0.1° grid cells to capture spatial variations. Information on point sources, such as power stations, was preferentially adopted whenever available [57], while provincial/county-level activity data were applied for other sources whenever possible. Various spatial proxies, such as population densities and gross domestic production, were used for the remaining sources. A time-for-space substitution method, previously developed, was used to simulate monthly variations in residential energy consumption [58]. Monthly resolved open-fire burning data were directly obtained from the literature [59]. By omitting intra-annual variation in emissions from other sources, monthly resolved total emissions were compiled.

### Decomposition of major drivers

Temporal trends in activity strengths, EFs and BC emissions were analysed to identify the impact of various socio-economic, technical and regulatory factors. The major drivers identified included changes in energy consumption and production outputs, the phasing-out of beehive coke ovens, the upgrading of brick/cement kilns, reduced on-road motor vehicle emissions caused by stricter emission standards, the promotion of end-of-pipe dust removal facilities for energy production and industry (e.g. coal pulverization, circulating fluidized bed and particulate matter abatement), rural-to-urban migration due to urbanization, residential energy-type switching and the upgrading of residential stoves. These drivers were quantified using structural decomposition analysis on an annual basis from 1960 to 2019 [10]. The results are presented as cumulative effects during this period and the accumulative net effects were calculated as the differences between the positive and negative contributions.

### Uncertainty analysis and data processing

Monte Carlo simulation was applied to analyse the uncertainty of the estimated emissions. The simulation was repeated for 10 000 runs by randomly drawing variables of activities and EFs from predefined frequency distributions. All activity intensities were assumed to follow uniform distributions, while the EFs were log-normally distributed. In fact, standard deviations were available for almost all EFs. However, only single numbers were available for all activity strengths. To generate a uniform distribution, a coefficient of variation of 10% was assumed for the consumption of residential wood, straw and charcoal, whereas a coefficient of variation of 5% was adopted for other sources [8]. It was also assumed that a certain percentage of installed end-of-pipe dust removal facilities failed to operate, with failure rates of 5% before 2005, declining linearly to 0% by 2010 [60]. The coefficient of variation for the failure rates was assumed to be 100%. All simulation results were presented as medians and semi-interquartile ranges. Data compilation and analysis were performed using Matlab R2019a and Excel 2019.

## Supplementary Material

nwae283_Supplemental_File

## Data Availability

Data associated with the study are available upon request.
