# Supplementary material for: Mitigating black carbon emissions: key drivers in residential usage and coke/brick production
Source: Natl Sci Rev. 2024 Aug 14;11(10):nwae283. doi: 10.1093/nsr/nwae283 (PMC11409891; doi:10.1093/nsr/nwae283)
Supplement: nwae283_Supplemental_File [file nwae283_supplemental_file.doc]

**Supporting Information for**

Mitigating Black Carbon Emissions: Key Drivers in Residential Usage and Coke/Brick Production

Jin Li^1^, Yuanzheng Zhang^1^, Shuxiu Zheng^1^, Jinghang Wang^1^, Rong Dai^1^, Wenxiao Zhang^1^, Haoran Xu^1^, Huizhong Shen^2^, Guofeng Shen^1^, Hefa Cheng^1^, Jianmin Ma^1^, Shu Tao^1,2^,*

*^1^ College of Urban and Environmental Sciences, Laboratory for Earth Surface Processes and Institute of Carbon Neutrality, Peking University, Beijing 100871, China.*

*^2^ School of Environmental Science and Engineering, Southern University of Science and Technology, Shenzhen 518055, China.*

* Corresponding author: Shu Tao

**Email:** [taos@pku.edu.cn](mailto:taos@pku.edu.cn)

**Figure S1** Comparison of temporal variations of total and sectorial BC emissions from this study and those from two previous publications [1,2].

**Table S1** List of BC emission sources quantified for a total of 146 detailed fuel/activity types in seven sectors. Various facilities/end-of-pipe mitigation techniques are shown whenever applicable.

| **Sector** | **Fuel/activity** | **Facility/End-of-**  **pipe mitigation**  **pipe mitigation** | **Ref*** | **Sector** | **Fuel/activity** | **Facility/End-of-**  **pipe mitigation** | **Ref*** |
| --- | --- | --- | --- | --- | --- | --- | --- |
| Power  genera-  tion | coal anthracite | PC-UN/CO;  ST-UN/CO;  CFB-UN/CO;  UL; | [3] | Residential | coal anthracite, CH | TR, IM, CL | [3–11] |
|  | coking coal |  |  |  | coking coal |  |  |
|  | bituminous |  |  |  | bituminous, CH |  |  |
|  | lignite |  |  |  | lignite |  |  |
|  | peat |  |  |  | peat |  |  |
|  | oil diesel |  |  |  | anthracite, BR |  |  |
|  | residue oil |  |  |  | bituminous, BR |  |  |
|  | crude oil |  |  |  | oil diesel |  |  |
|  | LPG |  |  |  | residue oil |  |  |
|  | gas natural gas |  |  |  | kerosene |  |  |
|  | waste municipal | ST-UN/CO;UL; |  |  | LPG |  |  |
|  | industrial |  |  |  | gas natural gas |  |  |
|  | biomass solid | ST-UN/CO;UL; |  |  | gas work gas |  |  |
|  | biogas |  |  |  | coke oven gas |  |  |
| Industrial  combus-  tion | coal anthracite | PC-UN/CO;  ST-UN/CO;  CFB-UN/CO; | [3, 12] |  | waste municipal | OP, TR, IM |  |
|  | coking coal |  |  |  | non-organized |  |  |
|  | bituminous |  |  |  | biomass wood log | OP, TR, IM, CL |  |
|  | lignite |  |  |  | tree branch |  |  |
|  | peat |  |  |  | grass | OP, TR, IM |  |
|  | coke |  |  |  | maize |  |  |
|  | oil diesel |  |  |  | corncob |  |  |
|  | residue oil |  |  |  | wheat |  |  |
|  | crude oil |  |  |  | rice |  |  |
|  | LPG |  |  |  | sugar cane |  |  |
|  | gas natural gas |  |  |  | soybean |  |  |
|  | gas flaring |  |  |  | cotton |  |  |
|  | gas works gas |  |  |  | other straws |  |  |
|  | coke oven gas |  |  |  | animal dung |  |  |
|  | blast furnace gas |  |  |  | pressed wood | TR, IM, CL |  |
|  | waste municipal | ST-UN/CO |  |  | pressed straw |  |  |
|  | industrial |  |  |  | charcoal | OP, TR, IM, CL |  |
|  | biomass solid | ST-UN/CO |  |  | biogas |  |  |
|  | biogas |  |  | Commercial | coal anthracite, CH |  | [3, 5] |
| Industrial processes | coking beehive |  | [13–19] |  | coking coal |  |  |
|  | mechanical | UN/CO |  |  | bituminous, CH |  |  |
|  | iron & sintering |  |  |  | lignite |  |  |
|  | steel ore pellet |  |  |  | peat |  |  |
|  | pig iron |  |  |  | anthracite, BR |  |  |
|  | electric arc |  |  |  | bituminous, BR |  |  |
|  | open hearth |  |  |  | oil diesel |  |  |
|  | O_2_ blown |  |  |  | residue fuel |  |  |
|  | hot rolling |  |  |  | kerosene |  |  |
|  | ferroalloy |  |  |  | LPG |  |  |
|  | petrol ammonia |  |  |  | gas natural gas |  |  |
|  | chemical fertilizer |  |  |  | gas work gas |  |  |
|  | catalytic cracking |  |  |  | coke oven |  |  |
|  | non-ferr. alumina |  |  |  | waste municipal |  |  |
|  | metals aluminum | SB/PB-UN/CO |  |  | industrial |  |  |
|  | lead | UN/CO |  |  | biomass wood log |  |  |
|  | magnesium |  |  |  | pressed wood |  |  |
|  | zinc |  |  |  | pressed straw |  |  |
|  | nickel |  |  |  | charcoal |  |  |
|  | copper |  |  |  | biogas |  |  |
|  | non- lime |  |  |  |  |  |  |
|  | metal | RK-UN/CO; |  |  |  |  |  |
|  | brick | TK-UN/CO; |  |  | | | |
|  |  | OK-UN/CO; |  | * major data sources | | | |
|  | cement crushing | UN/CO |  |  | | | |
|  | cement hydraulic | PK-UN/CO;  SK-UN/CO;  OK-UN/CO; |  |  |  |  |  |
|  | cement griding | UN/CO |  |  |  |  |  |
|  | glass |  |  |  | | | |
|  | mining natural gas |  |  |  |  |  |  |

**Table S1** List of BC emission sources quantified for a total of 146 detailed fuel/activity types in seven sectors. Various end-of-pipe mitigation techniques are shown whenever applicable (continued).

| **Sector** | **Fuel/activity** | **Facility/End-of-pipe mitigation** | **Ref*** | **Abbreviation** |
| --- | --- | --- | --- | --- |
| Transpor-  tation | coal rail, bituminous |  | [3, 14, 20] | BH beehive  BR briquettes  CF circulating fluidized bed  CH chunk  CL clean stove  CO controlled  cyclone  wet scrubber  electrostatic precipitator  fabric filter  IM improved stove  LNG liquefied natural gas  LPG liquefied petroleum gas  ME mechanical  NO no regulation  OK other kiln  OP open stove  PB pre baked electrode  PC pulverized coal furnace  PK precalciner kiln  RK rotary kiln  SB self baking electrode  SK shaft kiln  ST stoker coal furnace  TK tunnel kiln  TR traditional stove  UL ultra low  UN uncontrolled  * major data sources |
|  | oil rail, diesel |  |  |  |
|  | passenger vehicles, diesel | NO;  China I-VI; |  |  |
|  | other vehicles, diesel |  |  |  |
|  | small fishing boats inland, diesel |  |  |  |
|  | small fishing boats, ocean, diesel |  |  |  |
|  | other vessels, inland, diesel |  |  |  |
|  | other vessels, ocean, diesel |  |  |  |
|  | other vessels, ocean, residue oil |  |  |  |
|  | small fishing boats, ocean, residue oil |  |  |  |
|  | motorcycles/tricycles, gasoline |  |  |  |
|  | passenger vehicles, gasoline | NO;  China I-VI; |  |  |
|  | other vehicles, gasoline |  |  |  |
|  | domestic aviation, gasoline |  |  |  |
|  | domestic aviation, gasoline jet fuel |  |  |  |
|  | domestic aviation, kerosene jet fuel |  |  |  |
|  | international aviation, kerosene jet fuel |  |  |  |
|  | other vehicles, LPG |  |  |  |
|  | gas vehicles, natural gas |  |  |  |
|  | vessels, ocean, LNG |  |  |  |
|  | pipeline, natural gas |  |  |  |
|  | biomass vehicles, biogas |  |  |  |
|  | vehicles, liquid biofuel |  |  |  |
| Agriculture | coal bituminous |  | [3, 21] |  |
|  | oil off-road vehicles, diesel |  |  |  |
|  | off-road vehicles, residue oil |  |  |  |
|  | gas natural gas |  |  |  |
|  | biomass deforestation |  |  |  |
|  | crop residue, open-fire | maize, rice,  wheat, others |  |  |
| Natural | coal peat fire |  | [21] |  |
|  | biomass boreal forest fire |  |  |  |
|  | temperate forest fire |  |  |  |
|  | grassland fire |  |  |  |

**Table S2** Major updating in the BC emission inventory compilation.

| **Category** | **Sector** | **Updating** |
| --- | --- | --- |
| Source | Commercial | - Residential and commercial sectors were fully separated [3, 5]. |
|  | Residential | - Honeycomb/Coal briquettes were divided into anthracite and bituminous [7]. - New sources of grass and firelighter, pressed wood, and pressed straw were added [7, 10]. - Straws were classified into maize (corn stalk), wheat, rice, sugar cane, soybean, cotton, and others based on crop production [22] and residue-to-crop ratios [11]. - Fugitive emissions from residential stove emissions were added for the first time [23]. |
|  | Power | - Both crude oil and LPG were added [3]. |
|  | Industry | - Coke oven coke, LPG, gas works gas, coke oven gas, and blast furnace gas were included [3]. - Industrial processes of hydraulic cement, iron and steel, glass, lime, alumina calcination, and natural gas production were added [3, 15, 16, 18]. - Fugitive emissions from brick/kiln production were added for the first time [24]. |
|  | Transportation | - Heavy-duty and light-duty vehicles were quantified separately [14]. - Aviation was classified into domestic and international [3]. - Inland and ocean vessels were separated [3]. - Small fishing vessels were distinguished as an independent source [3, 20]. - Fuel types consumed by vessels were classified by specific fuel types [3]. - Rail, motorcycle, and tricycle sources were added as individual sources [3]. |
|  | Agricultural | - Bituminous coal, residue fuel oil, and natural gas were added [3]. |
| Activity | Residential | - Nonorganized open-burned waste was calculated based on per-capita municipal waste output, waste recycle rates, and nonorganized open-burning proportions of municipal waste derived from previous studies [4, 6, 9]. |
|  | Industry | - Activity intensities of industrial processes were updated based on IEA [3], World Steel Association [15], United States Geological Survey [16], United Nations Statistics Division [18], and yearbooks [19]. |
|  | Others | - Energy consumptions of other sources were updated based on IEA [3], WorldBank [12], and various yearbooks [5]. |
| Improved facilities and end-of-pipe abatement | Power and industry | - Circulating fluidized bed boiler was included in power stations, and pulverized coal furnace was included in industry [25–27]. - Ultra-low emission power stations from 2014 were identified and categorized independently [28]. - Application rates of cyclone, wet scrubber, electrostatic precipitator, and fabric filter were quantified and updated [26, 27, 29–34]. |
|  | Transportation | - Motor vehicles adopting various emissions standards from none to China I–VI were adopted [35–39]. |
|  | Residential | - Fractions of major stove types, including open, traditional, improved, and clean stoves, were quantified individually [40]. EFs for various stove-fuel combinations were applied. |
| EFs | Industry | - EFs of industrial-scale coke production were updated based on newly reported on-site measurements [41–44]. - EFs of beehive coke ovens were calculated based on EFs of industrial-scale coke ovens and field-measured BC concentrations in the air at the production sites of both industrial-scale and beehive coke ovens [45]. |
|  | Transportation | - EFs of motor vehicles were updated based on national emission standards of various stages (none, China I–VI) and recently reported on-site measurements [46–108]. |
|  | Others | - The updated database for EFs was from 265 references, including many recently reported [23-25, 41–302]. |
| Spatial  disaggrega-  tion | Power | - A new database with locations and power-generation capacities was adopted [303]. |
|  | Industry | - Location and production information of iron and steel industry and CO_2_ emissions from oil refineries were adopted [304, 305]. |
|  | Residential | - An updated model for heating degree day was adopted to disaggregate heating energy consumption [306]. |
|  | Transportation | - Spatial distributions of CO_2_ emissions from rail, vehicles, aviation, and navigation obtained from EDGAR were adopted as proxies in spatial interpolation of transportation emissions [307]. |
|  | Others | - Provincial fuel consumption or industrial product outputs were updated based on newly released yearbooks [5, 19, 308]. |

**Table S3**. Annual black carbon emissions from seven sectors in China from 1960 to 2019. The results are shown as means and 50% uncertainty intervals (Gg/year)

| Year | Power generation | Industrial | Residential | Commercial | Transportation | Agricultural | Natural |
| --- | --- | --- | --- | --- | --- | --- | --- |
| 1960 | 1.93 (1.12-3.43) | 446 (203-1019) | 947 (803-1123) | 5.76 (4.57-7.48) | 13.9 (6.84-28.9) | 39.9 (28.8-58.9) | 16.5 (14.7-18.6) |
| 1961 | 2.65 (1.53-4.71) | 207 (101-459) | 934 (791-1110) | 5.87 (4.66-7.64) | 12.0 (5.99-25.0) | 44.5 (33.1-63.1) | 16.9 (15.1-19.0) |
| 1962 | 3.29 (1.88-5.90) | 86.4 (47.9-187) | 941 (796-1118) | 6.09 (4.83-7.92) | 11.5 (5.82-23.8) | 45.5 (34.0-64.4) | 18.1 (16.1-20.3) |
| 1963 | 3.82 (2.17-6.93) | 71.6 (41.5-156) | 964 (816-1146) | 6.41 (5.08-8.37) | 12.3 (6.24-25.0) | 47.1 (35.2-66.5) | 15.4 (13.7-17.3) |
| 1964 | 3.55 (2.00-6.50) | 72.0 (41.6-162) | 989 (837-1175) | 6.74 (5.36-8.79) | 14.2 (7.25-28.8) | 53.7 (40.0-75.9) | 18.1 (16.2-20.3) |
| 1965 | 3.64 (2.03-6.70) | 76.8 (45.0-172) | 1015 (859-1208) | 6.84 (5.42-8.93) | 16.4 (8.41-33.5) | 60.4 (44.9-85.3) | 18.0 (16.0-20.2) |
| 1966 | 4.21 (2.34-7.84) | 91.2 (52.7-204) | 1044 (883-1242) | 6.98 (5.53-9.13) | 17.6 (9.10-35.7) | 62.6 (46.5-88.9) | 15.4 (13.7-17.3) |
| 1967 | 4.80 (2.62-8.98) | 72.9 (43.4-157) | 1069 (904-1269) | 7.13 (5.65-9.35) | 16.9 (8.90-33.8) | 64.0 (47.7-90.3) | 16.8 (15.0-18.9) |
| 1968 | 5.43 (2.94-10.3) | 77.3 (45.4-171) | 1094 (927-1301) | 7.28 (5.77-9.59) | 16.7 (8.95-32.9) | 66.1 (49.4-92.9) | 19.4 (17.3-21.8) |
| 1969 | 6.17 (3.33-11.8) | 99.0 (57.5-217) | 1126 (952-1338) | 7.45 (5.88-9.82) | 18.6 (10.1-36.5) | 65.7 (48.6-93.6) | 15.5 (13.8-17.4) |
| 1970 | 6.97 (3.71-13.4) | 128 (72.3-280) | 1159 (980-1375) | 7.62 (6.02-10.1) | 21.5 (11.7-41.8) | 73.1 (53.8-105) | 18.3 (16.3-20.5) |
| 1971 | 7.55 (3.98-14.7) | 164 (90.0-364) | 1191 (1008-1414) | 7.81 (6.15-10.3) | 23.5 (13.0-45.4) | 76.9 (56.3-111) | 19.2 (17.1-21.5) |
| 1972 | 8.24 (4.30-16.2) | 176 (94.7-396) | 1222 (1034-1452) | 7.95 (6.26-10.6) | 26.6 (14.9-51.1) | 80.8 (58.7-117) | 14.7 (13.1-16.6) |
| 1973 | 8.89 (4.61-17.6) | 173 (93.9-395) | 1253 (1061-1488) | 8.14 (6.39-10.8) | 31.5 (17.5-60.6) | 98.1 (71.7-142) | 17.0 (15.2-19.1) |
| 1974 | 9.60 (4.96-19.1) | 173 (93.7-396) | 1279 (1082-1520) | 8.37 (6.56-11.2) | 33.3 (18.7-63.3) | 104 (75.7-151) | 17.4 (15.6-19.6) |
| 1975 | 10.4 (5.31-20.9) | 203 (109-455) | 1303 (1101-1551) | 8.55 (6.70-11.4) | 36.9 (20.7-70.6) | 108 (79.1-157) | 14.6 (13.0-16.4) |
| 1976 | 11.1 (5.63-22.5) | 224 (122-486) | 1321 (1117-1573) | 8.72 (6.83-11.6) | 35.8 (20.5-67.5) | 106 (77.3-155) | 16.3 (14.5-18.2) |
| 1977 | 11.6 (5.79-23.6) | 267 (144-577) | 1341 (1134-1597) | 8.90 (6.96-11.9) | 39.7 (22.6-74.8) | 109 (79.0-160) | 16.5 (14.7-18.5) |
| 1978 | 11.2 (5.58-23.0) | 315 (170-686) | 1354 (1144-1613) | 9.27 (7.25-12.4) | 37.2 (21.8-68.2) | 99.5 (70.5-150) | 20.1 (17.9-22.6) |
| 1979 | 12.0 (5.92-24.7) | 323 (178-701) | 1376 (1161-1641) | 9.77 (7.64-13.1) | 40.7 (24.6-73.1) | 99.4 (70.4-150) | 17.4 (15.5-19.6) |
| 1980 | 12.4 (6.10-25.6) | 366 (206-777) | 1399 (1180-1667) | 10.1 (7.90-13.5) | 39.5 (24.0-70.3) | 91.8 (65.4-138) | 16.8 (15.0-18.9) |
| 1981 | 12.4 (6.05-25.9) | 341 (195-722) | 1393 (1174-1664) | 11.3 (8.85-15.0) | 41.8 (25.5-74.7) | 91.4 (65.4-136) | 16.6 (14.8-18.6) |
| 1982 | 13.0 (6.27-27.1) | 375 (216-779) | 1380 (1163-1650) | 11.2 (8.75-14.9) | 43.6 (26.7-77.6) | 93.9 (68.0-137) | 14.6 (13.0-16.4) |
| 1983 | 13.9 (6.66-29.5) | 397 (228-832) | 1369 (1152-1640) | 11.9 (9.30-15.9) | 45.2 (27.9-79.6) | 99.3 (72.3-144) | 15.4 (13.7-17.2) |
| 1984 | 15.4 (7.30-32.6) | 462 (263-965) | 1365 (1145-1641) | 13.1 (10.3-17.3) | 47.2 (29.2-83.0) | 107 (77.8-155) | 14.1 (12.6-15.9) |
| 1985 | 16.9 (8.04-36.0) | 506 (290-1048) | 1321 (1103-1594) | 12.8 (10.1-17.1) | 49.0 (30.6-85.2) | 114 (84.5-161) | 18.3 (16.3-20.6) |
| 1986 | 18.2 (8.60-39.1) | 598 (342-1222) | 1275 (1060-1551) | 15.1 (11.9-20.0) | 51.0 (32.4-87.2) | 117 (86.7-166) | 16.9 (15.1-18.9) |
| 1987 | 19.9 (9.35-43.0) | 677 (386-1382) | 1246 (1031-1523) | 17.0 (13.4-22.5) | 51.6 (33.1-87.4) | 118 (87.1-168) | 16.4 (14.6-18.4) |
| 1988 | 21.6 (10.1-46.6) | 754 (430-1525) | 1236 (1018-1518) | 19.1 (15.0-25.3) | 54.7 (35.6-91.6) | 122 (90.1-175) | 16.1 (14.3-18.1) |
| 1989 | 22.8 (10.6-49.1) | 805 (451-1643) | 1217 (998-1498) | 21.0 (16.4-28.0) | 57.0 (37.3-94.6) | 114 (82.6-165) | 12.5 (11.1-14.0) |
| 1990 | 23.6 (11.0-51.3) | 819 (455-1698) | 1211 (992-1497) | 22.7 (17.9-30.2) | 57.1 (38.3-92.4) | 125 (87.6-191) | 17.0 (15.1-19.0) |
| 1991 | 24.9 (11.6-53.9) | 825 (463-1711) | 1190 (973-1476) | 25.6 (20.2-34.0) | 59.4 (40.9-93.1) | 128 (89.0-195) | 17.0 (15.2-19.1) |
| 1992 | 26.0 (12.1-56.6) | 919 (508-1945) | 1149 (936-1427) | 25.1 (19.6-33.5) | 62.9 (44.6-95.8) | 112 (76.4-177) | 13.4 (11.9-15.0) |
| 1993 | 27.1 (12.6-59.1) | 1119 (608-2389) | 1113 (905-1386) | 27.6 (21.5-37.1) | 66.7 (48.0-99.7) | 110 (73.9-177) | 15.9 (14.2-17.9) |
| 1994 | 27.4 (12.8-59.5) | 1305 (696-2808) | 1070 (868-1335) | 26.3 (20.5-35.5) | 69.9 (51.3-102) | 118 (80.0-187) | 17.2 (15.3-19.3) |
| 1995 | 28.0 (13.0-60.9) | 1705 (884-3681) | 1048 (848-1313) | 28.5 (22.2-38.5) | 73.9 (54.9-106) | 124 (83.7-199) | 16.2 (14.4-18.2) |
| 1996 | 29.6 (13.7-64.3) | 1659 (855-3603) | 1025 (829-1284) | 30.3 (23.7-40.6) | 88.4 (66.3-124) | 85.0 (57.9-135) | 16.1 (14.4-18.1) |
| 1997 | 25.9 (12.1-56.6) | 1616 (833-3522) | 983 (794-1234) | 32.4 (25.2-43.5) | 95.6 (73.3-131) | 130 (87.8-209) | 20.2 (18.0-22.6) |
| 1998 | 24.2 (11.3-53.0) | 1271 (655-2855) | 957 (772-1202) | 32.6 (25.5-43.6) | 111 (86.2-148) | 140 (91.8-230) | 29.3 (26.1-32.8) |
| 1999 | 22.9 (10.7-50.2) | 1031 (530-2393) | 930 (748-1171) | 32.5 (25.2-44.1) | 126 (99.1-166) | 131 (83.9-221) | 21.0 (18.6-23.6) |
| 2000 | 23.5 (10.9-51.9) | 845 (437-2023) | 901 (726-1135) | 32.4 (25.4-43.0) | 135 (106-177) | 67.1 (47.1-103) | 16.8 (15.0-18.9) |
| 2001 | 22.9 (10.6-50.6) | 864 (438-2108) | 885 (713-1114) | 33.5 (26.3-44.6) | 147 (116-191) | 68.9 (48.3-106) | 11.9 (10.5-13.4) |
| 2002 | 23.6 (11.0-52.1) | 848 (416-2104) | 859 (692-1083) | 36.6 (28.8-48.6) | 157 (123-206) | 73.4 (51.0-114) | 7.49 (6.69-8.38) |
| 2003 | 25.9 (12.0-57.2) | 927 (452-2324) | 822 (660-1040) | 42.8 (33.7-56.5) | 167 (129-224) | 90.7 (63.4-139) | 25.1 (22.2-28.3) |
| 2004 | 26.6 (12.3-59.6) | 751 (392-1906) | 785 (628-996) | 49.4 (39.0-64.9) | 174 (134-234) | 105 (71.9-166) | 20.6 (18.4-23.1) |
| 2005 | 25.9 (11.9-57.4) | 728 (408-1811) | 753 (604-955) | 57.7 (45.7-75.5) | 182 (139-248) | 112 (77.1-174) | 13.0 (11.7-14.5) |
| 2006 | 26.2 (12.1-59.1) | 612 (375-1549) | 725 (581-920) | 63.6 (50.4-82.8) | 190 (145-258) | 115 (79.0-179) | 16.0 (14.2-18.0) |
| 2007 | 25.3 (11.7-56.8) | 600 (378-1489) | 686 (548-873) | 67.9 (54.0-88.3) | 197 (149-268) | 119 (83.0-182) | 13.6 (12.2-15.3) |
| 2008 | 21.1 (9.71-48.0) | 542 (350-1326) | 660 (527-842) | 68.5 (54.3-89.4) | 197 (149-269) | 117 (83.3-176) | 19.4 (17.2-22.0) |
| 2009 | 20.1 (9.28-46.1) | 566 (376-1306) | 636 (508-810) | 77.0 (61.3-99.9) | 196 (148-267) | 121 (85.7-182) | 18.5 (16.5-20.8) |
| 2010 | 18.9 (8.76-43.7) | 561 (380-1189) | 617 (492-786) | 78.5 (62.2-102) | 195 (146-266) | 125 (87.3-190) | 14.8 (13.2-16.5) |
| 2011 | 20.2 (9.30-47.0) | 543 (380-1098) | 592 (472-753) | 89.9 (71.6-116) | 199 (148-273) | 128 (90.7-192) | 15.6 (13.9-17.5) |
| 2012 | 19.9 (9.12-46.4) | 541 (378-1064) | 559 (445-711) | 92.6 (73.6-120) | 211 (156-291) | 131 (92.9-199) | 13.8 (12.3-15.5) |
| 2013 | 19.9 (9.12-46.4) | 583 (408-1098) | 527 (419-673) | 85.5 (68.1-111) | 208 (153-287) | 144 (103-216) | 13.0 (11.6-14.7) |
| 2014 | 18.4 (8.43-43.5) | 559 (390-1008) | 500 (398-640) | 84.0 (66.8-109) | 203 (149-282) | 151 (108-227) | 16.0 (14.3-18.0) |
| 2015 | 15.2 (6.96-36.2) | 522 (363-903) | 465 (368-596) | 91.9 (73.1-119) | 195 (143-272) | 160 (115-237) | 12.5 (11.2-14.0) |
| 2016 | 12.3 (5.66-29.4) | 501 (348-820) | 426 (338-545) | 90.6 (72.3-117) | 183 (133-256) | 156 (112-233) | 10.1 (9.04-11.2) |
| 2017 | 9.89 (4.58-23.8) | 433 (306-687) | 393 (311-504) | 82.4 (65.5-107) | 171 (123-239) | 162 (115-242) | 9.89 (8.86-11.0) |
| 2018 | 8.41 (3.90-20.5) | 396 (288-632) | 358 (283-460) | 74.9 (59.4-97.5) | 159 (115-224) | 137 (96-209) | 9.31 (8.34-10.4) |
| 2019 | 6.61 (3.08-16.4) | 339 (252-538) | 328 (258-422) | 68.3 (54.2-89.2) | 136 (98.3-191) | 131 (91-201) | 8.43 (7.56-9.40) |

**SI References**

1. Wang R, Tao S, Wang W *et al.* Black carbon emissions in china from 1949 to 2050. *Environ Sci Technol* 2012;**46**:7595–603.

2. Xu H, Ren Y, Zhang W *et al.* Updated global black carbon emissions from 1960 to 2017: improvements, trends, and drivers. *Environ Sci Technol* 2021;**55**:7869–79.

3. International Energy Agency (IEA). IEA World Energy Statistics and Balances; http://www.oecd-ilibrary.org/statistics (accessed June 20, 2023).

4. Cogut A. Open Burning of Waste: A Global Health Disaster; R20 Regions of Climate Action. 2016.

5. Department of Energy Statistics, and National Bureau of Statistics of People’s Republic of China. *China Energy Statistical Yearbook 1986-2020*; China Statistics Press: Beijing, China, 1986-2020.

6. Hao W. Study on emission characteristics of air pollutants from open burning of municipal solid waste. 2019.

7. Shen G, Xiong R, Tian Y *et al.* Substantial transition to clean household energy mix in rural China. *Natl Sci Rev* 2022;**9**.

8. Tao S, Ru MY, Du W *et al.* Quantifying the rural residential energy transition in China from 1992 to 2012 through a representative national survey. *Nat Energy* 2018;**3**:567–73.

9. Wiedinmyer C, Yokelson RJ, Gullett BK. Global emissions of trace gases, particulate matter, and hazardous air pollutants from open burning of domestic waste. *Environ Sci Technol* 2014;**48**:9523–30.

10. Xu J, Liu S, Jiang Y *et al.* Policy analysis and implementation of biomass molding fuels industry in China. *Adv New Renew Energy* 2015;**3**:477–84.

11. Yevich R, Logan JA. An assessment of biofuel use and burning of agricultural waste in the developing world. *Glob Biogeochem Cycles* 2003;**17**.

12. The Work Bank. *Global Gas Flaring Tracker Report*; World Bank Publications, Washington D. C., USA, 2021. <https://www.worldbank.org/en/topic/extractiveindustries/publication/2023-global-gas-flaring-tracker-report>

13. Xu Y, Shen H, Yun X *et al.* Health effects of banning beehive coke ovens and implementation of the ban in China. *Proc Natl Acad Sci USA* 2018;**115**:2693–8.

14. International Institute for Applied Systems Analysis (IIASA). *The Greenhouse Gas - Air Pollution Interaction and Synergies (GAINS) Model*; [https://gains.iiasa.ac.at](https://gains.iiasa.ac.at/) (accessed June 20, 2023).

15. World Steel Association (WSA). *Steel Statistic Yearbooks*; [https://worldsteel.org](https://worldsteel.org/) (accessed June 20, 2023).

16. United States Geological Survey (USGS). *Commodity Statistics and Information*; <https://www.usgs.gov/centers/national-minerals-information-center/commodity-statistics-and-information> (accessed June 20, 2023).

17. Nation Master. <https://www.nationmaster.com/>. Accessed 22 June, 2023.

18. United Nations Statistics Division (UNSD). *Industrial Commodity Statistics Database*; [https://data.un.org](https://data.un.org/) (accessed June 20, 2023).

19. China Building Materials Federation. *Almanac of China Building Materials Industry, 1981-2020*; Almanac of China Building Materials Industry Press: Beijing, China, 1982-2020..

20. Ministry of Agriculture and Rural Affairs of the People’s Republic of China. *China Fishery Statistical Yearbook, 2006-2020*; China Agriculture Press: Beijing, China, 2006-2020.

21. van der Werf GR, Randerson JT, Giglio L *et al.* Global fire emissions estimates during 1997–2016. *Earth Syst Sci Data* 2017;**9**:697–720.

22. Food and Agriculture Organization of the United Nations (FAO). Statistics at FAO, Production Indices. [http://www.fao.org/faostat/en/#data](http://www.fao.org/faostat/en/#data) (accessed June 20, 2023).

23. Luo ZH, Zhang L, Li G *et al.* Evaluating co-emissions into indoor and outdoor air of EC, OC, and BC from in-home biomass burning. *Atmospheric Res* 2021;**248**.

24. Chen Y, Du W, Zhuo S *et al.* Stack and fugitive emissions of major air pollutants from typical brick kilns in China. *Environ Pollut* 2017;**224**:421–9.

25. Wang S, Zhao X, Li X *et al.* Emission characteristics of fine particles from grate firing boilers (in Chinese). *Environ Sci* 2009;**30**:963-968.

26. Wang SX, Zhao B, Cai SY *et al.* Emission trends and mitigation options for air pollutants in East Asia. *Atmospheric Chem Phys* 2014;**14**:6571–603.

27. Yue T. Study on temporal-spatial characteristics and abatement potential of air pollutants emission from industrial boilers of China. 2019.

28. Ministry of Ecology and Environment of the People’s Republic of China, National Development and Reform Commission, National Energy Administration. *Fully Implement the Ultra-low Emission and Energy-saving Renovation Plan for Coal-fired Power Plants*; 2015. <https://www.mee.gov.cn/gkml/hbb/bwj/201512/t20151215_319170.htm> (accessed June 21, 2023).

29. China National Environmental Monitoring Centre. *Annual Statistic Report on Environment in China*; <http://www.cnemc.cn/jcbg/zghjtjnb> (accessed June 26, 2023).

30. China Environmental Yearbook Editorial Broad. *China Environmental Yearbook 1989-2020*; China Environmental Yearbook Press: Beijing, China, 1989-2020.

31. China Electricity Council. *China Electricity Statistic Yearbook 2020-2021*; China Statistic Press: Beijing, China, 2020-2021.

32. China Research Society of Urban Development. *The Yearbook of China’s Cities 1985-2020*; China Urban Yearbook Press: Beijing, China, 1985-2020.

33. Wang X, Lei Y, Yan L *et al.* A unit-based emission inventory of SO_2_, NOx and PM for the Chinese iron and steel industry from 2010 to 2015. *Sci Total Environ* 2019;**676**:18–30.

34. Liu J, Tong D, Zheng Y *et al.* Carbon and air pollutant emissions from China’s cement industry 1990–2015: trends, evolution of technologies, and drivers. *Atmospheric Chem Phys* 2021;**21**:1627–47.

35. Ministry of Ecology and Environment of the People’s Republic of China; General Administration of Quality Supervision, Inspection and Quarantine of the People’s Republic of China. *Limits and Measurement Methods for Emissions from Light-duty Vehicles (China 6) (GB 18352.6-2016)*; China Environment Publishing Group: Beijing, China, 2016. <https://www.mee.gov.cn/ywgz/fgbz/bz/bzwb/dqhjbh/dqydywrwpfbz/201612/t20161223_369476.shtml> (accessed June 21, 2023).

36. Ministry of Ecology and Environment of the People’s Republic of China; State Administration for Market Regulation. *Limits and Measurement Methods for Emissions from Diesel Fueled Heavy-duty Vehicles (China VI) (GB 17691-2018)*; 2018. <https://www.mee.gov.cn/ywgz/fgbz/bz/bzwb/dqhjbh/dqydywrwpfbz/201807/t20180703_445995.shtml> (accessed June 21, 2023).

37. Ministry of Ecology and Environment of the People’s Republic of China; General Administration of Quality Supervision, Inspection and Quarantine of the People’s Republic of China. *Limits and Measurement Methods for Emissions from Light-duty Vehicles (China 5) (GB 18352.6-2013)*; China Environment Publishing Group: Beijing, China, 2013. <https://www.mee.gov.cn/ywgz/fgbz/bz/bzwb/dqhjbh/dqydywrwpfbz/201309/t20130917_260352.shtml> (accessed June 21, 2023).

38. Ministry of Ecology and Environment of the People’s Republic of China; General Administration of Quality Supervision, Inspection and Quarantine of the People’s Republic of China. *Limits and Measurement Methods for Emissions from Light-duty Vehicles (Ⅲ, Ⅳ) (GB 18352.3-2005)*; China Environment Publishing Group: Beijing, China, 2005. <https://www.mee.gov.cn/ywgz/fgbz/bz/bzwb/dqhjbh/dqydywrwpfbz/200707/t20070701_66145.shtml> (accessed June 21, 2023).

39. Ministry of Ecology and Environment of the People’s Republic of China; General Administration of Quality Supervision, Inspection and Quarantine of the People’s Republic of China. *Limits and Measurement Methods for Emissions from Light-duty Vehicles (Ⅰ) (GB 18352.1-2001)*; China Environment Publishing Group: Beijing, China, 2001. <https://www.mee.gov.cn/ywgz/fgbz/bz/bzwb/dqhjbh/dqydywrwpfbz/200104/t20010416_67420.shtml> (accessed June 21, 2023).

40. Meng W, Shen G, Shen H *et al.* Synergistic health benefits of household stove upgrading and energy switching in rural China. *Environ Sci Technol* 2021;**55**:14567–75.

41. Kupiainen K, Klimont Z. *Primary Emissions of Submicron and Carbonaceous Particles in Europe and the Potential for Their Control*. Laxenburg, Austria: International Institute for Applied Systems Analysis, 2004.

42. Mu L, Li X, Liu X *et al.* Characterization and emission factors of carbonaceous aerosols originating from coke production in China. *Environ Pollut* 2021;**268**.

43. Jing D, Mu L, Wang J *et al.* Characterization of carbon contents in fly ash from coking processes (in Chinese). *China Environ Sci* 2017;**37**:4097-4102.

44. Mu L. Rearch on emission characteristics of main atmospheric pollution and their behavior during mechanical coking processes. 2013.

45. He Q, Fan X, Wang X *et al.* Emissions of TSP and sulfur dioxide (SO_2_) from coal coking process. *Earth Environ* 2007;**35**:279–83.

46. Wang B, Lau Y-S, Huang Y *et al.* Chemical and toxicological characterization of particulate emissions from diesel vehicles. *J Hazard Mater* 2021;**405**.

47. Kostenidou E, Martinez-Valiente A, R’Mili B *et al.* Technical note: Emission factors, chemical composition, and morphology of particles emitted from Euro 5 diesel and gasoline light-duty vehicles during transient cycles. *Atmospheric Chem Phys* 2021;**21**:4779–96.

48. Hao Y, Deng S, Qiu Z *et al.* Chemical characterization of PM_2.5_ emitted from China IV and China V light-duty vehicles in China. *Sci Total Environ* 2021;**783**.

49. Tang R, Tan R, Wang H *et al.* Physical and chemical characterization of particle emissions from gasoline direct injection vehicle and its influencing factors (in Chinese). *Acta Sci Circumstantiae* 2020;**40**:846-853.

50. Zhao X, Wang J, Zhu S *et al.* Emission characteristics of exhaust PM and Its carbonaceous components from China Ⅲ to China Ⅳ diesel vehicles in Shenyang (in Chinese). *Environ Sci* 2019;**40**:4330-4336.

51. Yang JC, Roth P, Zhu HW *et al.* Impacts of gasoline aromatic and ethanol levels on the emissions from GDI vehicles: Part 2. Influence on particulate matter, black carbon, and nanoparticle emissions. *Fuel* 2019;**252**:812–20.

52. Yang H-H, Dhital NB, Wang L-C *et al.* Chemical characterization of fine particulate matter in gasoline and diesel vehicle exhaust. *Aerosol Air Qual Res* 2019;**19**:1439–49.

53. Song J, He L, Hu J *et al.* Real-world emission characteristics of China Ⅱ heavy-duty diesel trucks with different payloads (in Chinese). *Environ Pollut Control* 2019;**41**:34-40.

54. Hu H, Zhang J, Liu X *et al.* Study on the emission characteristics of carbon components from motor vehicles’ exhaust particulate matter. *Annual Conference of Science and Technology of Chinese Society For Environmental Sciences*. China Environmental Science Press, 2019, 1113–9.

55. Shen X, Wu H, Cao X *et al.* Gaseous and carbonaceous composition of PM_2.5_ emitted from rural vehicles in China. *Aerosol Air Qual Res* 2018;**18**:1993–2004.

56. Ma C, Zhuang T, Zhang Z *et al.* Tailpipe emission characteristics of PM_2.5_ from selected on-road China III and China IV diesel vehicles. *Aerosol Sci Technol* 2018;**52**:799–808.

57. Huang C, Hu Q-Y, Lu J. Measurements of OC and EC emission factors for light-duty gasoline vehicles (in Chinese). *Environ Sci* 2018;**39**:3110-3117.

58. Du Z, Hu M, Peng J *et al.* Comparison of primary aerosol emission and secondary aerosol formation from gasoline direct injection and port fuel injection vehicles. *Atmospheric Chem Phys* 2018;**18**:9011–23.

59. Zavala M, Molina LT, Yacovitch TI *et al.* Emission factors of black carbon and co-pollutants from diesel vehicles in Mexico City. *Atmospheric Chem Phys* 2017;**17**:15293–305.

60. Saliba G, Saleh R, Zhao Y *et al.* Comparison of gasoline direct-unjection (GDI) and port fuel injection (PFI) vehicle emissions: emission certification standards, cold-Start, secondary organic aerosol formation potential, and potential climate impacts. *Environ Sci Technol* 2017;**51**:6542–52.

61. Platt SM, El Haddad I, Pieber SM *et al.* Gasoline cars produce more carbonaceous particulate matter than modern filter-equipped diesel cars. *Sci Rep* 2017;**7**.

62. Jaiprakash, Habib G. Chemical and optical properties of PM_2.5_ from on-road operation of light duty vehicles in Delhi city. *Sci Total Environ* 2017;**586**:900–16.

63. Hays MD, Preston W, George BJ *et al.* Temperature and driving cycle significantly affect carbonaceous gas and particle matter emissions from diesel trucks. *Energy Fuels* 2017;**31**:11034–42.

64. Cai T, Zhang Y, Fang D *et al.* Chinese vehicle emissions characteristic testing with small sample size: Results and comparison. *Atmospheric Pollut Res* 2017;**8**:154–63.

65. Qin Y, Hu M, Li M *et al.* Physical and chemical characteristics of PM_2.5_ emissions from gasoline direct injection engine and its influence factors (in Chinese). *China Environ Sci* 2016;**36**:1332-1339.

66. Kim Y, Sartelet K, Seigneur C *et al.* Effect of measurement protocol on organic aerosol measurements of exhaust emissions from gasoline and diesel vehicles. *Atmos Environ* 2016;**140**:176–87.

67. Huang C, Lou S, Qiao L *et al.* Physicochemical characteristics of real-world PM emissions from heavy-duty diesel buses (in Chinese). *Res Environ Sci* 2016;**29**:1352-1361.

68. Chen J, Fan W, Li Y *et al.* Characteristics of PM_2.5_ emissions and its carbonaceous components analysis from China Ⅲ diesel vehicles in Chengdu (in Chinese). *Environ Eng* 2016;**34**:546-550+576.

69. Zhang Y, Yao Z, Shen X *et al.* Chemical characterization of PM_2.5_ emitted from on-road heavy-duty diesel trucks in China. *Atmos Environ* 2015;**122**.

70. Wu B, Shen X, Cao X *et al.* Carbonaceous composition of PM_2.5_ emitted from on-road China III diesel trucks in Beijing, China. *Atmos Environ* 2015;**116**:216–24.

71. Wang G, Lang J, Cheng S *et al.* Characteristics of PM_2.5_ and hydrocarbon emitted from heavy-duty diesel vehicle (in Chinese). *China Environ Sci* 2015;**35**:3581-3587.

72. He L, Hu J, Zu L *et al.* Emission characteristics of exhaust PM_2.5_ and its carbonaceous components from China Ⅰ to China Ⅲ heavy-duty diesel vehicles. *Acta Sci Circumstantiae* 2015;**35**:656–62.

73. Alves CA, Lopes DJ, Calvo AI *et al.* Emissions from light-duty diesel and gasoline in-use vehicles measured on chassis dynamometer test cycles. *Aerosol Air Qual Res* 2015;**15**:99–116.

74. May AA, Nguyen NT, Presto AA *et al.* Gas- and particle-phase primary emissions from in-use, on-road gasoline and diesel vehicles. *Atmos Environ* 2014;**88**:247–60.

75. Dallmann TR, Onasch TB, Kirchstetter TW *et al.* Characterization of particulate matter emissions from on-road gasoline and diesel vehicles using a soot particle aerosol mass spectrometer. *Atmospheric Chem Phys* 2014;**14**:7585–99.

76. Platt SM, El Haddad I, Zardini AA *et al.* Secondary organic aerosol formation from gasoline vehicle emissions in a new mobile environmental reaction chamber. *Atmospheric Chem Phys* 2013;**13**:9141–58.

77. Dallmann TR, Kirchstetter TW, DeMartini SJ *et al.* Quantifying on-road emissions from gasoline-powered motor vehicles: accounting for the presence of medium- and heavy-duty diesel trucks. *Environ Sci Technol* 2013;**47**:13873–81.

78. Mancilla Y, Mendoza A. A tunnel study to characterize PM_2.5_ emissions from gasoline-powered vehicles in Monterrey, Mexico. *Atmos Environ* 2012;**59**:449–60.

79. Chiang H-L, Lai Y-M, Chang S-Y. Pollutant constituents of exhaust emitted from light-duty diesel vehicles. *Atmos Environ* 2012;**47**:399–406.

80. Oanh NTK, Thiansathit W, Bond TC *et al.* Compositional characterization of PM_2.5_ emitted from in-use diesel vehicles. *Atmos Environ* 2010;**44**:15–22.

81. Dwyer H, Ayala A, Zhang S *et al.* A study of emissions from a Euro 4 light duty diesel vehicle with the European particulate measurement programme. *Atmos Environ* 2010;**44**:3469–76.

82. Subramanian R, Winijkul E, Bond TC *et al.* Climate-relevant properties of diesel particulate emissions: results from a piggyback study in Bangkok, Thailand. *Environ Sci Technol* 2009;**43**:4213–8.

83. Cheung KL, Polidori A, Ntziachristos L *et al.* Chemical characteristics and oxidative potential of particulate matter emissions from gasoline, diesel, and biodiesel cars. *Environ Sci Technol* 2009;**43**:6334–40.

84. Biswas S, Verma V, Schauer JJ *et al.* Chemical speciation of PM emissions from heavy-duty diesel vehicles equipped with diesel particulate filter (DPF) and selective catalytic reduction (SCR) retrofits. *Atmos Environ* 2009;**43**:1917–25.

85. Schneider J, Kirchner U, Borrmann S *et al.* In situ measurements of particle number concentration, chemically resolved size distributions and black carbon content of traffic-related emissions on German motorways, rural roads and in city traffic. *Atmos Environ* 2008;**42**:4257–68.

86. Schauer JJ, Christensen CG, Kittelson DB *et al.* Impact of ambient temperatures and driving conditions on the chemical composition of particulate matter emissions from non-smoking gasoline-powered motor vehicles. *Aerosol Sci Technol* 2008;**42**:210–23.

87. Ning Z, Polidori A, Schauer JJ *et al.* Emission factors of PM species based on freeway measurements and comparison with tunnel and dynamometer studies. *Atmos Environ* 2008;**42**:3099–114.

88. He L, Hu M, Zhang Y *et al.* Fine particle emissions from on-road vehicles in the Zhujiang Tunnel, China. *Environ Sci Technol* 2008;**42**:4461–6.

89. Ban-Weiss GA, McLaughlin JP, Harley RA *et al.* Long-term changes in emissions of nitrogen oxides and particulate matter from on-road gasoline and diesel vehicles. *Atmos Environ* 2008;**42**:220–32.

90. Shah SD, Cocker DR, Johnson KC *et al.* Reduction of particulate matter emissions from diesel backup generators equipped with four different exhaust aftertreatment devices. *Environ Sci Technol* 2007;**41**:5070–6.

91. Robert MA, Kleeman MJ, Jakober CA. Size and composition distributions of particulate matter emissions: Part 2- Heavy-duty diesel vehicles. *J Air Waste Manag Assoc* 2007;**57**:1429–38.

92. Fujita EM, Zielinska B, Campbell DE *et al.* Variations in speciated emissions from spark-ignition and compression-ignition motor vehicles in California’s south coast air basin. *J Air Waste Manag Assoc* 2007;**57**:705–20.

93. Michael D. Geller, Leonidas Ntziachristos, Athanasios Mamakos *et al.* Physicochemical and redox characteristics of particulate matter (PM) emitted from gasoline and diesel passenger cars. *Atmos Environ* 2006;**40**.

94. Grieshop AP, Lipsky EM, Pekney NJ *et al.* Fine particle emission factors from vehicles in a highway tunnel: Effects of fleet composition and season. *Atmos Environ* 2006;**40**:S287–98.

95. Lough GC, Schauer JJ, Park JS *et al.* Emissions of metals associated with motor vehicle roadways. *Environ Sci Technol* 2005;**39**:826–36.

96. Geller VD, Sardar SB, Phuleria H *et al.* Measurements of particle number and mass concentrations and size distributions in a tunnel environment. *Environ Sci Technol* 2005;**39**:8653–63.

97. Zielinska B, Sagebiel J, McDonald JD *et al.* Emission rates and comparative chemical composition from selected in-use diesel and gasoline-fueled vehicles. *J Air Waste Manag Assoc* 2004;**54**:1138–50.

98. Shah SD, Cocker DR, Miller JW *et al.* Emission rates of particulate matter and elemental and organic carbon from in-use diesel engines. *Environ Sci Technol* 2004;**38**:2544–50.

99. Cocker DR, Shah SD, Johnson KC *et al.* Development and application of a mobile laboratory for measuring emissions from diesel engines. 2. Sampling for toxics and particulate matter. *Environ Sci Technol* 2004;**38**:6809–16.

100. Alander TJA, Leskinen AP, Raunemaa TM *et al.* Characterization of diesel particles: Effects of fuel reformulation, exhaust aftertreatment, and engine operation on particle carbon composition and volatility. *Environ Sci Technol* 2004;**38**:2707–14.

101. Schauer JJ, Kleeman MJ, Cass GR *et al.* Measurement of emissions from air pollution sources. 5. C-1-C-32 organic compounds from gasoline-powered motor vehicles. *Environ Sci Technol* 2002;**36**:1169–80.

102. Cadle SH, Mulawa P, Groblicki P *et al.* In-use light-duty gasoline vehicle particulate matter emissions on three driving cycles. *Environ Sci Technol* 2001;**35**:26–32.

103. Allen JO, Mayo PR, Hughes LS *et al.* Emissions of size-segregated aerosols from on-road vehicles in the Caldecott Tunnel. *Environ Sci Technol* 2001;**35**:4189–97.

104. Schauer JJ, Kleeman MJ, Cass GR *et al.* Measurement of emissions from air pollution sources. 2. C-1 through C-30 organic compounds from medium duty diesel trucks. *Environ Sci Technol* 1999;**33**:1578–87.

105. Kirchstetter TW, Harley RA, Kreisberg NM *et al.* On-road measurement of fine particle and nitrogen oxide emissions from light- and heavy-duty motor vehicles. *Atmos Environ* 1999;**33**:2955–68.

106. Norbeck JM, Durbin TD, Truex TJ. *Measurement of Primary Particulate Matter Emissions from Light-Duty Motor Vehicles*. Center for Environmental Research and Technology, College of Engineering, University of California, Riverside, CA, 1998.

107. Sagebiel JC, Zielinska B, Walsh PA *et al.* PM-10 exhaust samples collected during IM-240 dynamometer tests of in-service vehicles in Nevada. *Environ Sci Technol* 1997;**31**:75–83.

108. Williams DJ, Milne JW, Quigley SM *et al.* Particulate emissions from “in-use” motor vehicles - II. Diesel vehicles. *Atmos Environ* 1989;**23**:2647–61.

109. Bond TC, Streets DG, Yarber KF *et al.* A technology-based global inventory of black and organic carbon emissions from combustion. *J Geophys Res-Atmospheres* 2004;**109**:D14203.

110. Zeng X, Kong S, Zhang Q *et al.* Source profiles and emission factors of organic and inorganic species in fine particles emitted from the ultra-low emission power plant and typical industries. *Sci Total Environ* 2021;**789**:147966–147966.

111. Liu Y, Yan J, Xu W *et al.* Emission characteristics of conventional air pollutants in coal-fired power plants after ultra-low emission transformation (in Chinese). *Acta Sci Circumstantiae* 2020;**40**:1967-1975.

112. Jin M. Study on atmospheric pollutants emission characteristicsand mitigation potential of aluminum industrial furnace in Zhengzhou city. 2020.

113. Hu Z, Wang X, Zhang L *et al.* Emission characteristics of particulate matters from a 30 MW biomass-fired power plant in China. *Renew Energy* 2020;**155**:225–36.

114. Chen X, Liu Q, Yuan C *et al.* Emission characteristics of fine particulate matter from ultra-low emission power plants. *Environ Pollut* 2019;**255**.

115. Zavala M, Molina LT, Maiz P *et al.* Black carbon, organic carbon, and co-pollutant emissions and energy efficiency from artisanal brick production in Mexico. *Atmospheric Chem Phys* 2018;**18**:6023–37.

116. Xu J, Huang C, Li L *et al.* Chemical composition characteristics of PM_2.5_ emitted by medium and small capacity coal-fired boilers in the Yangtze River Delta region (in Chinese). *Environ Sci* 2018;**39**:1493-1501.

117. He K, Zhang Q, Wang S. Technical manual for the compilation of urban air pollutant emission inventory made by Tsinghua University. 2018.

118. Klimont Z, Kupiainen K, Heyes C *et al.* Global anthropogenic emissions of particulate matter including black carbon. *Atmospheric Chem Phys* 2017;**17**:8681–723.

119. Guo Y, Gao X, Zhu T *et al.* Chemical profiles of PM emitted from the iron and steel industry in northern China. *Atmos Environ* 2017;**150**:187–97.

120. Weyant C, Athalye V, Ragavan S *et al.* Emissions from South Asian brick production. *Environ Sci Technol* 2014;**48**:6477–83.

121. Zhao K. Research on emission characteristics and environmental impacts of the particulate matter emitted from a coal-fired power plant in Peking. 2013.

122. Shang Y. Research on layer burning industrial boiler _PM2.5_ emissions characteristics. 2012.

123. Lei Y, Zhang Q, He KB *et al.* Primary anthropogenic aerosol emission trends for China, 1990-2005. *Atmospheric Chem Phys* 2011;**11**:931–54.

124. Sippula O, Hokkinen J, Puustinen H *et al.* Comparison of particle emissions from small heavy fuel oil and wood-fired boilers. *Atmos Environ* 2009;**43**:4855–64.

125. Li C, Li X, Duan L *et al.* Emission characteristics of PM_10_ from coal-fired industrial boiler (in Chinese). *Environ Sci* 2009;**30**:650-655.

126. Zhang Y, Schauer JJ, Zhang Y *et al.* Characteristics of particulate carbon emissions from real-world Chinese coal combustion. *Environ Sci Technol* 2008;**42**:5068–73.

127. Zhou N, Zeng L, Yu X *et al.* The design and field test of a dilution tunnel for stationary sources (in Chinese). *Acta Sci Circumstantiae* 2006;**26**:764-772.

128. Reddy MS, Venkataraman C. Inventory of aerosol and sulphur dioxide emissions from India: I - Fossil fuel combustion. *Atmos Environ* 2002;**36**:677–97.

129. Ge S, Bai ZP, Liu WL *et al.* Boiler briquette coal versus raw coal: Part I - Stack gas emissions. *J Air Waste Manag Assoc* 2001;**51**:524–33.

130. Wang L, Du W, Chen Y *et al.* High PM_2.5_ emission from typical old, small fishing vessels in China. *Environ Sci Technol Lett* 2022;**9**:199–204.

131. Zhang F, Chen Y, Su P *et al.* Variations and characteristics of carbonaceous substances emitted from a heavy fuel oil ship engine under different operating loads. *Environ Pollut* 2021;**284**.

132. Zhao J, Zhang Y, Yang Z *et al.* A comprehensive study of particulate and gaseous emissions characterization from an ocean-going cargo vessel under different operating conditions. *Atmos Environ* 2020;**223**.

133. Zhang F, Guo H, Chen YJ *et al.* Size-segregated characteristics of organic carbon (OC), elemental carbon (EC) and organic matter in particulate matter (PM) emitted from different types of ships in China. *Atmospheric Chem Phys* 2020;**20**:1549–64.

134. Wu Z, Zhang Y, He J *et al.* Dramatic increase in reactive volatile organic compound (VOC) emissions from ships at berth after implementing the fuel switch policy in the Pearl River Delta Emission Control Area. *Atmospheric Chem Phys* 2020;**20**:1887–900.

135. Shen F, Li X. Effects of fuel types and fuel sulfur content on the characteristics of particulate emissions in marine low-speed diesel engine. *Environ Sci Pollut Res* 2020;**27**:37229–36.

136. Corbin JC, Peng W, Yang J *et al.* Characterization of particulate matter emitted by a marine engine operated with liquefied natural gas and diesel fuels. *Atmos Environ* 2020;**220**.

137. Zhou S, Zhou JX, Zhu YQ. Chemical composition and size distribution of particulate matters from marine diesel engines with different fuel oils. *Fuel* 2019;**235**:972–83.

138. Zhang F, Chen Y, Cui M *et al.* Emission factors and environmental implication of organic pollutants in PM emitted from various vessels in China. *Atmos Environ* 2019;**200**:302–11.

139. Xiao X, Li C, Ye X *et al.* Emission characteristics of gas-and particle-phase pollutants from river vessels in cruising mode (in Chinese). *Acta Sci Circumstantiae* 2019;**39**:13-24.

140. Zhang F, Chen Y, Chen Q *et al.* Real-world emission factors of gaseous and particulate pollutants from marine fishing boats and their total emissions in China. *Environ Sci Technol* 2018;**52**:4910–9.

141. Huang C, Hu Q, Wang H *et al.* Emission factors of particulate and gaseous compounds from a large cargo vessel operated under real-world conditions. *Environ Pollut* 2018;**242**:667–74.

142. Huang X, Zhang Z, Yang W *et al.* Emission factors and preliminary emission estimates of air pollutants from ships at berth in the Guangzhou port (in Chinese). *Environ Sci* 2017;**38**:3162-3168.

143. Peng Z, Ge Y, Tan J *et al.* Emissions from several in-use ships tested by portable emission measurement system. *Ocean Eng* 2016;**116**:260–7.

144. Gysel NR, Russell RL, Welch WA *et al.* Impact of aftertreatment technologies on the in-use gaseous and particulate matter emissions from a tugboat. *Energy Fuels* 2016;**30**:684–9.

145. Radischat C, Sippula O, Stengel B *et al.* Real-time analysis of organic compounds in ship engine aerosol emissions using resonance-enhanced multiphoton ionisation and proton transfer mass spectrometry. *Anal Bioanal Chem* 2015;**407**:5939–51.

146. Mueller L, Jakobi G, Czech H *et al.* Characteristics and temporal evolution of particulate emissions from a ship diesel engine. *Appl Energy* 2015;**155**:204–17.

147. Sippula O, Stengel B, Sklorz M *et al.* Particle emissions from a marine engine: chemical composition and aromatic emission profiles under various operating conditions. *Environ Sci Technol* 2014;**48**:11721–9.

148. Cappa CD, Williams EJ, Lack DA *et al.* A case study into the measurement of ship emissions from plume intercepts of the NOAA ship Miller Freeman. *Atmospheric Chem Phys* 2014;**14**:1337–52.

149. Moldanova J, Fridell E, Winnes H *et al.* Physical and chemical characterisation of PM emissions from two ships operating in European Emission Control Areas. *Atmospheric Meas Tech* 2013;**6**:3577–96.

150. Diesch JM, Drewnick F, Klimach T *et al.* Investigation of gaseous and particulate emissions from various marine vessel types measured on the banks of the Elbe in Northern Germany. *Atmospheric Chem Phys* 2013;**13**:3603–18.

151. Khan MY, Giordano M, Gutierrez J *et al.* Benefits of two mitigation strategies for container vessels: cleaner engines and cleaner fuels. *Environ Sci Technol* 2012;**46**:5049–56.

152. Petzold A, Lauer P, Fritsche U *et al.* Operation of marine diesel engines on biogenic fuels: modification of emissions and resulting climate effects. *Environ Sci Technol* 2011;**45**:10394–400.

153. Lack DA, Cappa CD, Langridge J *et al.* Impact of fuel quality regulation and speed reductions on shipping emissions: implications for climate and air quality. *Environ Sci Technol* 2011;**45**:9052–60.

154. Jayaram V, Nigam A, Welch WA *et al.* Effectiveness of emission control technologies for auxiliary engines on ocean-going vessels. *J Air Waste Manag Assoc* 2011;**61**:14–21.

155. Jayaram V, Agrawal H, Welch WA *et al.* Real-time gaseous, PM and ultrafine particle emissions from a modern marine engine operating on biodiesel. *Environ Sci Technol* 2011;**45**:2286–92.

156. Petzold A, Weingartner E, Hasselbach I *et al.* Physical properties, chemical composition, and cloud forming potential of particulate emissions from a marine diesel engine at various load conditions. *Environ Sci Technol* 2010;**44**:3800–5.

157. Agrawal H, Welch WA, Henningsen S *et al.* Emissions from main propulsion engine on container ship at sea. *J Geophys Res-Atmospheres* 2010;**115**.

158. Murphy SM, Agrawal H, Sorooshian A *et al.* Comprehensive simultaneous shipboard and airborne characterization of exhaust from a modern container ship at sea. *Environ Sci Technol* 2009;**43**:4626–40.

159. Moldanova J, Fridell E, Popovicheva O *et al.* Characterisation of particulate matter and gaseous emissions from a large ship diesel engine. *Atmos Environ* 2009;**43**:2632–41.

160. Lack DA, Corbett JJ, Onasch T *et al.* Particulate emissions from commercial shipping: Chemical, physical, and optical properties. *J Geophys Res-Atmospheres* 2009;**114**.

161. Agrawal H, Sawant AA, Jansen K *et al.* Characterization of chemical and particulate emissions from aircraft engines. *Atmos Environ* 2008;**42**:4380–92.

162. Agrawal H, Malloy QGJ, Welch WA *et al.* In-use gaseous and particulate matter emissions from a modern ocean going container vessel. *Atmos Environ* 2008;**42**:5504–10.

163. Petzold A, Schroder FP. Jet engine exhaust aerosol characterization. *Aerosol Sci Technol* 1998;**28**:62–76.

164. Zhang B, Shen Z, Sun J *et al.* Emission characteristics and formation mechanisms of PM_2.5_ and gases from different geological maturities coals combustion. *Fuel* 2022;**315**.

165. Mandal TK, Yadav L, Sharma SK *et al.* Chemical properties of emissions from solid residential fuels used for energy in the rural sector of the southern region of India. *Environ Sci Pollut Res* 2022;**29**:37930–53.

166. Cui M, Xu Y, Yu B *et al.* Characterization of carbonaceous substances emitted from residential solid fuel combustion using real-world data from the Beijing-Tianjin-Hebei region. *Sci Total Environ* 2022;**837**.

167. Zhang L, Luo Z, Li Y *et al.* Optically measured black and particulate brown carbon emission factors from real-world residential combustion predominantly affected by fuel fifferences. *Environ Sci Technol* 2021;**55**:169–78.

168. Zhang B, Sun J, Jiang N *et al.* Emission factors, characteristics, and gas-particle partitioning of polycyclic aromatic hydrocarbons in PM_2.5_ emitted for the typical solid fuel combustions in rural Guanzhong Plain, China. *Environ Pollut* 2021;**286**.

169. Wang J, Zhang S, Chen Y *et al.* Field based measurement of multiple pollutant emissions from residential coal burning in rural Shanxi, northern China. *Atmospheric Pollut Res* 2021;**12**:443–50.

170. Li XH, Yang KQ, Wang ZH *et al.* Theoretical equilibration time is supported by measurement study of residence time at dilution sampling on fine particulate matter emissions from household biofuel burning. *Chemosphere* 2021;**267**.

171. Islam MM, Wathore R, Zerriffi H *et al.* In-use emissions from biomass and LPG stoves measured during a large, multi-year cookstove intervention study in rural India. *Sci Total Environ* 2021;**758**.

172. Zhang Y, Shen Z, Zhang B *et al.* Carbonaceous aerosols emission reduction by using red mud additive in coal briquette. *Fuel Process Technol* 2020;**199**.

173. Liu C. Study on source profile of particulate matter emissionsfrom typical domestic combustion sources. 2020.

174. Cheng K, Hao W, Wang Y *et al.* Understanding the emission pattern and source contribution of hazardous air pollutants from open burning of municipal solid waste in China. *Environ Pollut* 2020;**263**.

175. Zhang Y, Zhang D, Liu Y *et al.* Design and application of one portable field measurement system for testing pollutant emissions from household stoves (in Chinese). *Renew Energy Resour* 2019;**37**:1751-1756.

176. Xu T, Li J, Chen N *et al.* Emission characteristics of PM_2.5_ from domestic biomass in rural areas of Wuhan (in Chinese). *J Jianghan Univ Nat Sci Ed* 2019;**47**:345-350.

177. Weyant CL, Thompson R, Lam NL *et al.* In-field emission measurements from biogas and liquified petroleum gas (LPG) stoves. *Atmosphere* 2019;**10**.

178. Weyant CL, Chen P, Vaidya A *et al.* Emission measurements from traditional biomass cookstoves in South Asia and Tibet. *Environ Sci Technol* 2019;**53**:3306–14.

179. Vicente ED, Vicente AM, Evtyugina M *et al.* Emissions from residential pellet combustion of an invasive acacia species. *Renew Energy* 2019;**140**:319–29.

180. van Zyl L, Tryner J, Bilsback KR *et al.* Effects of fuel moisture content on emissions from a rocket-elbow cookstove. *Environ Sci Technol* 2019;**53**:4648–56.

181. Thompson RJ, Li J, Weyant CL *et al.* Field emission measurements of solid fuel stoves in Yunnan, China demonstrate dominant causes of uncertainty in household emission inventories. *Environ Sci Technol* 2019;**53**:3323–30.

182. Sun J, Shen Z, Zhang Y *et al.* Effects of biomass briquetting and carbonization on PM_2.5_ emission from residential burning in Guanzhong Plain, China. *Fuel* 2019;**244**:379–87.

183. Pervez S, Verma M, Tiwari S *et al.* Household solid fuel burning emission characterization and activity levels in India. *Sci Total Environ* 2019;**654**:493–504.

184. Padilla-Barrera Z, Torres-Jardon R, Gerardo Ruiz-Suarez L *et al.* Determination of emission factors for climate forcers and air pollutants from improved wood-burning cookstoves in Mexico. *Energy Sustain Dev* 2019;**50**:61–8.

185. Liu Y, Zhong L, Yan J *et al.* Carbon compositions and VOCs emission characteristics of civil combustion fuels (in Chinese). *China Environ Sci* 2019;**39**:1412-1418.

186. Li Q, Qi J, Jiang J *et al.* Significant reduction in air pollutant emissions from household cooking stoves by replacing raw solid fuels with their carbonized products. *Sci Total Environ* 2019;**650**:653–60.

187. Johnson MA, Garland CR, Jagoe K *et al.* In-home emissions performance of cookstoves in Asia and Africa. *Atmosphere* 2019;**10**.

188. Hays MD, Kinsey J, George I *et al.* Carbonaceous particulate matter emitted from a pellet-fired biomass boiler. *Atmosphere* 2019;**10**.

189. de la Sota C, Viana M, Kane M *et al.* Quantification of carbonaceous aerosol emissions from cookstoves in Senegal. *Aerosol Air Qual Res* 2019;**19**:80–91.

190. Das D, Bhandarkar U, Sethi V. Influence of the Inclusion of Ignition Stage Emissions in the Development of Emission Factors for Coal Cookstoves Used in India. *Environ Sci Technol* 2019;**53**:3149–56.

191. Czaplicka M, Cieslik E, Komosinski B *et al.* Emission factors for biofuels and coal combustion in a domestic boiler of 18 kW. *Atmosphere* 2019;**10**.

192. Champion WM, Grieshop AP. Pellet-fed gasifier stoves approach gas-stove like performance during in-home use in Rwanda. *Environ Sci Technol* 2019;**53**:6570–9.

193. Yang G, Kong S, Zheng S *et al.* Size-resolved emission factors of carbonaceous particles from domestic coal combustion in China (in Chinese). *Environ Sci* 2018;**39**:3524-3534.

194. Xie M, Shen G, Holder AL *et al.* Light absorption of organic carbon emitted from burning wood, charcoal, and kerosene in household cookstoves. *Environ Pollut* 2018;**240**:60–7.

195. Wang Y, Hao W, Cheng K *et al.* Emission characteristics and chemical components of PM_2.5_ from open burning of municipal solid waste (in Chinese). *Environ Sci* 2018;**39**:3518-3523.

196. Tian J, Ni H, Han Y *et al.* Primary PM_2.5_ and trace gas emissions from residential coal combustion: assessing semi-coke briquette for emission reduction in the Beijing-Tianjin-Hebei region, China. *Atmos Environ* 2018;**191**:378–86.

197. Sun J, Zhi G, Jin W *et al.* Emission factors of organic carbon and elemental carbon for residential coal and biomass fuels in China- A new database for 39 fuel-stove combinations. *Atmos Environ* 2018;**190**:241–8.

198. Sun J, Shen Z, Zhang L *et al.* Impact of primary and secondary air supply intensity in stove on emissions of size-segregated particulate matter and carbonaceous aerosols from apple tree wood burning. *Atmospheric Res* 2018;**202**:33–9.

199. Shen G, Hays MD, Smith KR *et al.* Evaluating the performance of household liquefied petroleum gas cookstoves. *Environ Sci Technol* 2018;**52**:904–15.

200. Ozgen S, Caserini S. Methane emissions from small residential wood combustion appliances: Experimental emission factors and warming potential. *Atmos Environ* 2018;**189**:164–73.

201. Klauser F, Carlon E, Kistler M *et al.* Emission characterization of modern wood stoves under real-life oriented operating conditions. *Atmos Environ* 2018;**192**:257–66.

202. Keita S, Liousse C, Yoboue V *et al.* Particle and VOC emission factor measurements for anthropogenic sources in West Africa. *Atmospheric Chem Phys* 2018;**18**:7691–708.

203. Jayarathne T, Stockwell CE, Bhave PV *et al.* Nepal Ambient Monitoring and Source Testing Experiment (NAMaSTE): emissions of particulate matter from wood- and dung-fueled cooking fires, garbage and crop residue burning, brick kilns, and other sources. *Atmospheric Chem Phys* 2018;**18**:2259–86.

204. Eilenberg SR, Bilsback KR, Johnson M *et al.* Field measurements of solid-fuel cookstove emissions from uncontrolled cooking in China, Honduras, Uganda, and India. *Atmos Environ* 2018;**190**:116–25.

205. Du W, Zhu X, Chen Y *et al.* Field-based emission measurements of biomass burning in typical Chinese built-in-place stoves. *Environ Pollut* 2018;**242**:1587–97.

206. Wathore R, Mortimer K, Grieshop AP. In-use emissions and estimated impacts of traditional, natural- and forced-draft cookstoves in rural Malawi. *Environ Sci Technol* 2017;**51**:1929–38.

207. Tian J, Ni H, Cao J *et al.* Characteristics of carbonaceous particles from residential coal combustion and agricultural biomass burning in China. *Atmospheric Pollut Res* 2017;**8**:521–7.

208. Sun J, Shen Z, Cao J *et al.* Particulate matters emitted from maize straw burning for winter heating in rural areas in Guanzhong Plain, China: Current emission and future reduction. *Atmospheric Res* 2017;**184**:66–76.

209. Shen X, Guo Z, Jiang W *et al.* Carbon characteristics and elemental carbon isotopic compositions in biomass indoor combustion products (in Chinese). *China Environ Sci* 2017;**37**:3669-3674.

210. Reece SM, Sinha A, Grieshop AP. Primary and photochemically aged aerosol emissions from biomass cookstoves: chemical and physical characterization. *Environ Sci Technol* 2017;**51**:9379–90.

211. Pandey A, Patel S, Pervez S *et al.* Aerosol emissions factors from traditional biomass cookstoves in India: insights from field measurements. *Atmospheric Chem Phys* 2017;**17**:13721–9.

212. Nystrom R, Lindgren R, Avagyan R *et al.* Influence of wood species and burning conditions on particle emission characteristics in a residential wood stove. *Energy Fuels* 2017;**31**:5514–24.

213. Garland C, Delapena S, Prasad R *et al.* Black carbon cookstove emissions: A field assessment of 19 stove/fuel combinations. *Atmos Environ* 2017;**169**:140–9.

214. Du W, Shen G, Chen Y *et al.* Comparison of air pollutant emissions and household air quality in rural homes using improved wood and coal stoves. *Atmos Environ* 2017;**166**:215–23.

215. Coffey ER, Muvandimwe D, Hagar Y *et al.* New emission factors and efficiencies from in-field measurements of traditional and improved cookstoves and their potential implications. *Environ Sci Technol* 2017;**51**:12508–17.

216. Champion WM, Connors L, Montoya LD. Emission factors of fine particulate matter, organic and elemental carbon, carbon monoxide, and carbon dioxide for four solid fuels commonly used in residential heating by the US Navajo Nation. *J Air Waste Manag Assoc* 2017;**67**:1020–35.

217. Xu Y, Wang Y, Chen Y *et al.* Characterization of fine and carbonaceous particles emissions from pelletized biomass-coal blends combustion: Implications on residential crop residue utilization in China. *Atmos Environ* 2016;**141**:312–9.

218. Tian J. Laboratory study on emission characteristics of PM_2.5_ from crop residue burning and residential coal combustion in China. 2016.

219. Saxena M, Sharma SK, Tomar N *et al.* Residential biomass burning emissions over Northwestern Himalayan region of India: chemical characterization and budget estimation. *Aerosol Air Qual Res* 2016;**16**:504–18.

220. Magnone E, Park S-K, Park JH. Effects of moisture contents in the common oak on carbonaceous aerosols generated from combustion processes in an indoor wood stove. *Combust Sci Technol* 2016;**188**:982–96.

221. Li Q, Li X, Jiang J *et al.* Semi-coke briquettes: towards reducing emissions of primary PM_2.5_, particulate carbon, and carbon monoxide from household coal combustion in China. *Sci Rep* 2016;**6**.

222. Li Q, Jiang J, Zhang Q *et al.* Influences of coal size, volatile matter content, and additive on primary particulate matter emissions from household stove combustion. *Fuel* 2016;**182**:780–7.

223. Chen Y, Shen G, Su S *et al.* Efficiencies and pollutant emissions from forced-draft biomass-pellet semi-gasifier stoves: Comparison of International and Chinese water boiling test protocols. *Energy Sustain Dev* 2016;**32**:22–30.

224. Chen Y, Shen G, Liu W *et al.* Field measurement and estimate of gaseous and particle pollutant emissions from cooking and space heating processes in rural households, northern China. *Atmos Environ* 2016;**125**:265–71.

225. Vicente ED, Duarte MA, Tarelho LAC *et al.* Particulate and gaseous emissions from the combustion of different biofuels in a pellet stove. *Atmos Environ* 2015;**120**:15–27.

226. Shen G, Chen Y, Xue C *et al.* Pollutant emissions from improved coal- and wood-fuelled cookstoves in rural households. *Environ Sci Technol* 2015;**49**:6590–8.

227. Chen Y, Tian C, Feng Y *et al.* Measurements of emission factors of PM_2.5_, OC, EC, and BC for household stoves of coal combustion in China. *Atmos Environ* 2015;**109**:190–6.

228. Calvo AI, Martins V, Nunes T *et al.* Residential wood combustion in two domestic devices: Relationship of different parameters throughout the combustion cycle. *Atmos Environ* 2015;**116**:72–82.

229. Arora P, Jain S. Estimation of organic and elemental carbon emitted from wood burning in traditional and improved cookstoves using controlled cooking test. *Environ Sci Technol* 2015;**49**:3958–65.

230. Zhang H, Zhu T, Wang S *et al.* Indoor emissions of carbonaceous aerosol and other air pollutants from household fuel burning in Southwest China. *Aerosol Air Qual Res* 2014;**14**:1779-U291.

231. Wei S, Shen G, Zhang Y *et al.* Field measurement on the emissions of PM, OC, EC and PAHs from indoor crop straw burning in rural China. *Environ Pollut* 2014;**184**:18–24.

232. Tang X, Huang C, Lou S *et al.* Emission factors and PM chemical composition study of biomass burning in the Yangtze River Delta region (in Chinese). *Environ Sci* 2014;**35**:1623-1632.

233. Sen A, Mandal TK, Sharma SK *et al.* Chemical properties of emission from biomass fuels used in the rural sector of the western region of India. *Atmos Environ* 2014;**99**:411–24.

234. Kong S, Bai Z, Lu B. Comparative analysis on emission factors of carbonaceous components in PM_2.5_ and PM_10_ from domestic fuels combustion (in Chinese). *China Environ Sci* 2014;**34**:2749-2756.

235. Wei S, Su Y, Shen G *et al.* Emission factors of particulate matter and elemental carbon from rural residential wood combustion (in Chinese). *Asian J Ecotoxicol* 2013;**8**:29-36.

236. Shen G, Xue M, Wei S *et al.* Influence of fuel moisture, charge size, feeding rate and air ventilation conditions on the emissions of PM, OC, EC, parent PAHs, and their derivatives from residential wood combustion. *J Environ Sci* 2013;**25**:1808–16.

237. Shen G, Xue M, Wei S *et al.* Influence of fuel mass load, oxygen supply and burning rate on emission factor and size distribution of carbonaceous particulate matter from indoor corn straw burning. *J Environ Sci* 2013;**25**:511–9.

238. Shen G, Tao S, Wei S *et al.* Field measurement of emission factors of PM, EC, OC, Parent, nitro-, and oxy- polycyclic aromatic hydrocarbons for residential briquette, coal cake, and wood in rural Shanxi, China. *Environ Sci Technol* 2013;**47**:2998–3005.

239. Saud T, Saxena M, Singh DP *et al.* Spatial variation of chemical constituents from the burning of commonly used biomass fuels in rural areas of the Indo-Gangetic Plain (IGP), India. *Atmos Environ* 2013;**71**:158–69.

240. Just B, Rogak S, Kandlikar M. Characterization of ultrafine particulate matter from traditional and improved biomass cookstoves. *Environ Sci Technol* 2013;**47**:3506–12.

241. Shen G, Wei S, Wei W *et al.* Emission factors, size distributions, and emission inventories of carbonaceous particulate matter from residential wood combustion in rural China. *Environ Sci Technol* 2012;**46**:4207–14.

242. Shen G, Tao S, Wei S *et al.* Reductions in emissions of carbonaceous particulate matter and polycyclic aromatic hydrocarbons from combustion of biomass pellets in comparison with raw fuel burning. *Environ Sci Technol* 2012;**46**:6409–16.

243. Saud T, Gautam R, Mandal TK *et al.* Emission estimates of organic and elemental carbon from household biomass fuel used over the Indo-Gangetic Plain (IGP), India. *Atmos Environ* 2012;**61**:212–20.

244. Orasche J, Seidel T, Hartmann H *et al.* Comparison of emissions from wood combustion. part 1: emission factors and characteristics from different small-scale residential heating appliances considering particulate matter and polycyclic aromatic hydrocarbon (PAH)-related toxicological potential of particle-bound organic species. *Energy Fuels* 2012;**26**:6695–704.

245. Schmidl C, Luisser M, Padouvas E *et al.* Particulate and gaseous emissions from manually and automatically fired small scale combustion systems. *Atmos Environ* 2011;**45**:7443–54.

246. Lamberg H, Nuutinen K, Tissari J *et al.* Physicochemical characterization of fine particles from small-scale wood combustion. *Atmos Environ* 2011;**45**:7635–43.

247. Goncalves C, Alves C, Fernandes AP *et al.* Organic compounds in PM_2.5_ emitted from fireplace and woodstove combustion of typical Portuguese wood species. *Atmos Environ* 2011;**45**:4533–45.

248. Fernandes AP, Alves CA, Goncalves C *et al.* Emission factors from residential combustion appliances burning Portuguese biomass fuels. *J Environ Monit* 2011;**13**:3196–206.

249. Alves C, Goncalves C, Fernandes AP *et al.* Fireplace and woodstove fine particle emissions from combustion of western Mediterranean wood types. *Atmospheric Res* 2011;**101**:692–700.

250. Shen G, Yang Y, Wang W *et al.* Emission factors of particulate matter and elemental carbon for crop residues and coals burned in typical household stoves in China. *Environ Sci Technol* 2010;**44**:7157–62.

251. Goncalves C, Alves C, Evtyugina M *et al.* Characterisation of PM_10_ emissions from woodstove combustion of common woods grown in Portugal. *Atmos Environ* 2010;**44**:4474–80.

252. Christian TJ, Yokelson RJ, Cardenas B *et al.* Trace gas and particle emissions from domestic and industrial biofuel use and garbage burning in central Mexico. *Atmospheric Chem Phys* 2010;**10**:565–84.

253. Zhi G, Peng C, Chen Y *et al.* Deployment of coal briquettes and improved stoves: possibly an option for both environment and climate. *Environ Sci Technol* 2009;**43**:5586–91.

254. Roden CA, Bond TC, Conway S *et al.* Laboratory and field investigations of particulate and carbon monoxide emissions from traditional and improved cookstoves. *Atmos Environ* 2009;**43**:1170–81.

255. Li X, Wang S, Duan L *et al.* Carbonaceous aerosol emissions from household biofuel combustion in China. *Environ Sci Technol* 2009;**43**:6076–81.

256. Zhi G, Chen Y, Feng Y *et al.* Emission characteristics of carbonaceous particles from various residential coal-stoves in China. *Environ Sci Technol* 2008;**42**:3310–5.

257. MacCarty N, Ogle D, Still D *et al.* A laboratory comparison of the global warming impact of five major types of biomass cooking stoves. *Energy Sustain Dev* 2008;**12**:56–65.

258. Kleeman MJ, Robert MA, Riddle SG *et al.* Size distribution of trace organic species emitted from biomass combustion and meat charbroiling. *Atmos Environ* 2008;**42**:3059–75.

259. Habib G, Venkataraman C, Bond TC *et al.* Chemical, microphysical and optical properties of primary particles from the combustion of biomass fuels. *Environ Sci Technol* 2008;**42**:8829–34.

260. Cao G, Zhang X, Gong S *et al.* Investigation on emission factors of particulate matter and gaseous pollutants from crop residue burning. *J Environ Sci* 2008;**20**:50–5.

261. Liu Y, Zhang Y, Wei Y *et al.* Measurement of emission factors of carbonaceous aerosols from residential coal combustion. *Acta Sci Circumstantiae* 2007;**27**:1409–16.

262. Roden CA, Bond TC, Conway S *et al.* Emission factors and real-time optical properties of particles emitted from traditional wood burning cookstoves. *Environ Sci Technol* 2006;**40**:6750–7.

263. Chen Y, Zhi G, Feng Y *et al.* Measurements of emission factors for primary carbonaceous particles from residential raw-coal combustion in China. *Geophys Res Lett* 2006;**33**.

264. Venkataraman C, Habib G, Eiguren-Fernandez A *et al.* Residential biofuels in South Asia: Carbonaceous aerosol emissions and climate impacts. *Science* 2005;**307**:1454–6.

265. Parashar DC, Gadi R, Mandal TK *et al.* Carbonaceous aerosol emissions from India. *Atmos Environ* 2005;**39**:7861–71.

266. Chen Y, Sheng G, Bi X *et al.* Emission factors for carbonaceous particles and polycyclic aromatic hydrocarbons from residential coal combustion in China. *Environ Sci Technol* 2005;**39**:1861–7.

267. Fine PM, Cass GR, Simoneit BRT. Chemical characterization of fine particle emissions from the wood stove combustion of prevalent United States tree species. *Environ Eng Sci* 2004;**21**:705–21.

268. Fine PM, Cass GR, Simoneit BRT. Chemical characterization of fine particle emissions from the fireplace combustion of wood types grown in the Midwestern and Western United States. *Environ Eng Sci* 2004;**21**:387–409.

269. Fine PM, Cass GR, Simoneit BRT. Chemical characterization of fine particle emissions from the fireplace combustion of woods grown in the Southern United States. *Environ Sci Technol* 2002;**36**:1442–51.

270. Schauer JJ, Kleeman MJ, Cass GR *et al.* Measurement of emissions from air pollution sources. 3. C-1-C-29 organic compounds from fireplace combustion of wood. *Environ Sci Technol* 2001;**35**:1716–28.

271. Fine PM, Cass GR, Simoneit BRT. Chemical characterization of fine particle emissions from fireplace combustion of woods grown in the northeastern United States. *Environ Sci Technol* 2001;**35**:2665–75.

272. Andreae MO, Merlet P. Emission of trace gases and aerosols from biomass burning. *Glob Biogeochem Cycles* 2001;**15**:955–66.

273. McDonald JD, Zielinska B, Fujita EM *et al.* Fine particle and gaseous emission rates from residential wood combustion. *Environ Sci Technol* 2000;**34**:2080–91.

274. Hildemann LM, Markowski GR, Cass GR. Chemical composition of emissions from urban sources of fine organic aerosol. *Environ Sci Technol* 1991;**25**:744–59.

275. Dasch JM. Particulate and gaseous emissions from wood-burning fireplaces. *Environ Sci Technol* 1982;**16**:639–49.

276. Cooper JA. Environmental impact of residential wood combustion emissions and its implications. *J Air Pollut Control Assoc* 1980;**30**:855–61.

277. Santiago-De la Rosa N, Mugica-Alvarez V, Gonzalez-Cardoso G *et al.* Emission factors of polycyclic aromatic hydrocarbons and oxidative potential of fine particles emitted from crop residues burning. *Polycycl Aromat Compd* 2021;**42**:5123–42.

278. Wang Y, Hu M, Xu N *et al.* Chemical composition and light absorption of carbonaceous aerosols emitted from crop residue burning: influence of combustion efficiency. *Atmospheric Chem Phys* 2020;**20**:13721–34.

279. Wang Y, Hao W, Cheng K *et al.* Emission factors of typical air pollutants from open burning of crop straws (in Chinese). *China Environ Sci* 2018;**38**:2055-2061.

280. Mugica-Alvarez V, Hernandez-Rosas F, Magana-Reyes M *et al.* Sugarcane burning emissions: Characterization and emission factors. *Atmos Environ* 2018;**193**:262–72.

281. Zhang H, Hu J, Qi Y *et al.* Emission characterization, environmental impact, and control measure of PM_2.5_ emitted from agricultural crop residue burning in China. *J Clean Prod* 2017;**149**:629–35.

282. Fang Z, Deng W, Zhang Y *et al.* Open burning of rice, corn and wheat straws: primary emissions, photochemical aging, and secondary organic aerosol formation. *Atmospheric Chem Phys* 2017;**17**:14821–39.

283. Wang Z, Tan J, Bi X *et al.* Emission characteristics and chemical species from agricultural straw burning smoke (in Chinese). *Environ Sci Technol* 2016;**39**:150-155.

284. Tian J, Chow JC, Cao J *et al.* A biomass combustion chamber: design, evaluation, and a case study of wheat straw combustion emission tests. *Aerosol Air Qual Res* 2015;**15**:2104–14.

285. Ni H, Han Y, Cao J *et al.* Emission characteristics of carbonaceous particles and trace gases from open burning of crop residues in China. *Atmos Environ* 2015;**123**:399–406.

286. Hayashi K, Ono K, Kajiura M *et al.* Trace gas and particle emissions from open burning of three cereal crop residues: Increase in residue moistness enhances emissions of carbon monoxide, methane, and particulate organic carbon. *Atmos Environ* 2014;**95**:36–44.

287. Zhang Y, Shao M, Lin Y *et al.* Emission inventory of carbonaceous pollutants from biomass burning in the Pearl River Delta Region, China. *Atmos Environ* 2013;**76**:189–99.

288. Hall D, Wu C-Y, Hsu Y-M *et al.* PAHs, carbonyls, VOCs and PM_2.5_ emission factors for pre-harvest burning of Florida sugarcane. *Atmos Environ* 2012;**55**:164–72.

289. Vicente A, Alves C, Monteiro C *et al.* Measurement of trace gases and organic compounds in the smoke plume from a wildfire in Penedono (central Portugal). *Atmos Environ* 2011;**45**:5172–82.

290. Oanh NTK, Ly BT, Tipayarom D *et al.* Characterization of particulate matter emission from open burning of rice straw. *Atmos Environ* 2011;**45**:493–502.

291. Alves CA, Vicente A, Monteiro C *et al.* Emission of trace gases and organic components in smoke particles from a wildfire in a mixed-evergreen forest in Portugal. *Sci Total Environ* 2011;**409**:1466–75.

292. Akagi SK, Yokelson RJ, Wiedinmyer C *et al.* Emission factors for open and domestic biomass burning for use in atmospheric models. *Atmospheric Chem Phys* 2011;**11**:4039–72.

293. van der Werf GR, Randerson JT, Giglio L *et al.* Global fire emissions and the contribution of deforestation, savanna, forest, agricultural, and peat fires (1997-2009). *Atmospheric Chem Phys* 2010;**10**:11707–35.

294. McMeeking GR, Kreidenweis SM, Baker S *et al.* Emissions of trace gases and aerosols during the open combustion of biomass in the laboratory. *J Geophys Res-Atmospheres* 2009;**114**.

295. Mcmeeking GR. The optical, chemical, and physical properties of aerosols and gases emitted by the laboratory combustion of wildland fuels. 2008.

296. Sahai S, Sharma C, Singh DP *et al.* A study for development of emission factors for trace gases and carbonaceous particulate species from in situ burning of wheat straw in agricultural fields in india. *Atmos Environ* 2007;**41**:9173–86.

297. Li X, Wang S, Duan L *et al.* Particulate and trace gas emissions from open burning of wheat straw and corn stover in China. *Environ Sci Technol* 2007;**41**:6052–8.

298. Keshtkar H, Ashbaugh LL. Size distribution of polycyclic aromatic hydrocarbon particulate emission factors from agricultural burning. *Atmos Environ* 2007;**41**:2729–39.

299. Ranil Dhammapala, Candis Claiborn, Jorge Jimenez *et al.* Emission factors of PAHs, methoxyphenols, levoglucosan, elemental carbon and organic carbon from simulated wheat and Kentucky bluegrass stubble burns. *Atmos Environ* 2006;**41**.

300. Hays MD, Fine PM, Geron CD *et al.* Open burning of agricultural biomass: Physical and chemical properties of particle-phase emissions. *Atmos Environ* 2005;**39**:6747–64.

301. Christian TJ, Kleiss B, Yokelson RJ *et al.* Comprehensive laboratory measurements of biomass-burning emissions: 1. Emissions from Indonesian, African, and other fuels. *J Geophys Res-Atmospheres* 2003;**108**.

302. Ferek RJ, Reid JS, Hobbs PV *et al.* Emission factors of hydrocarbons, halocarbons, trace gases and particles from biomass burning in Brazil. *J Geophys Res-Atmospheres* 1998;**103**:32107–18.

303. Byers L, Friedrich J, Hennig R *et al.* A Global Database of Power Plants. Washington DC, World Resources Institute. 2019. <https://www.wri.org/research/global-database-power-plants>. Accessed July 12, 2023.

304. Carbon Emission Accounts & Datasets (CEADs). <https://www.ceads.net.cn/data/> (accessed June 25, 2023).

305. Lei T, Guan D, Shan Y *et al.* Adaptive CO2 emissions mitigation strategies of global oil refineries in all age groups. *One Earth* 2021;**4**:1114–26.

306. Chen H, Huang Y, Shen H *et al.* Modeling temporal variations in global residential energy consumption and pollutant emissions. *Appl Energy* 2016;**184**:820–9.

307. European Commission, Joint Research Centre (JRC)/Netherlands Environmental Assessment Agency (PBL). *Emissions Database for Global Atmospheric Research, release version 6.1 (EDGAR_v6.1)*; [https://edgar.jrc.ec.europa.eu/dataset_ap61#p1](https://edgar.jrc.ec.europa.eu/dataset_ap61#p1) (accessed June 24, 2023).

308. National Bureau of Statistics of the People’s Republic of China. *China Statistics Yearbook 1981-2020*; China Statistics Press: Beijing, China, 1981-2020.
